# Screening inducers of neuronal BDNF gene transcription using primary cortical cell cultures from BDNF-luciferase transgenic mice

**DOI:** 10.1038/s41598-019-48361-4

**Published:** 2019-08-14

**Authors:** Mamoru Fukuchi, Yui Okuno, Hironori Nakayama, Aoi Nakano, Hisashi Mori, Satoru Mitazaki, Yuka Nakano, Kazufumi Toume, Michiko Jo, Ichiro Takasaki, Kazuki Watanabe, Naotoshi Shibahara, Katsuko Komatsu, Akiko Tabuchi, Masaaki Tsuda

**Affiliations:** 1 Laboratory of Molecular Neuroscience, Faculty of Pharmacy, Takasaki University of Health and Welfare, 60 Nakaorui-machi, Takasaki-shi, Gunma 370-0033 Japan; 2 Department of Biological Chemistry, Graduate School of Medicine and Pharmaceutical Sciences, University of Toyama, 2630 Sugitani, Toyama-shi, Toyama 930-0194 Japan; 3 Department of Molecular Neuroscience, Graduate School of Medicine and Pharmaceutical Sciences, University of Toyama, 2630 Sugitani, Toyama-shi, Toyama 930-0194 Japan; 4 Laboratory of Forensic Toxicology, Faculty of Pharmacy, Takasaki University of Health and Welfare, 60 Nakaorui-machi, Takasaki-shi, Gunma 370-0033 Japan; 5 Division of Pharmacognosy, Institute of Natural Medicine, University of Toyama, 2630 Sugitani, Toyama-shi, Toyama 930-0194 Japan; 6 Division of Kampo Diagnostics, Institute of Natural Medicine, University of Toyama, 2630 Sugitani, Toyama-shi, Toyama 930-0194 Japan; 7 Department of Pharmacology, Graduate School of Science and Engineering, University of Toyama, 3190 Gofuku, Toyama-shi, Toyama 930-8555 Japan; 8 Laboratory of Natural Medicines, Faculty of Pharmacy, Takasaki University of Health and Welfare, 60 Nakaorui-machi, Takasaki-shi, Gunma 370-0033 Japan

**Keywords:** Neurotrophic factors, Neurochemistry

## Abstract

Brain-derived neurotrophic factor (BDNF) is a key player in synaptic plasticity, and consequently, learning and memory. Because of its fundamental role in numerous neurological functions in the central nervous system, BDNF has utility as a biomarker and drug target for neurodegenerative and neuropsychiatric disorders. Here, we generated a screening assay to mine inducers of *Bdnf* transcription in neuronal cells, using primary cultures of cortical cells prepared from a transgenic mouse strain, specifically, *Bdnf-Luciferase* transgenic (*Bdnf-Luc*) mice. We identified several active extracts from a library consisting of 120 herbal extracts. In particular, we focused on an active extract prepared from Ginseng Radix (GIN), and found that GIN activated endogenous *Bdnf* expression via cAMP-response element-binding protein-dependent transcription. Taken together, our current screening assay can be used for validating herbal extracts, food-derived agents, and chemical compounds for their ability to induce *Bdnf* expression in neurons. This method will be beneficial for screening of candidate drugs for ameliorating symptoms of neurological diseases associated with reduced *Bdnf* expression in the brain, as well as candidate inhibitors of aging-related cognitive decline.

## Introduction

Brain-derived neurotrophic factor (BDNF), a member of the neurotrophin family, is a key molecule in synaptic plasticity and related phenomena such as long-term memory^1^. Because of its fundamental role in neural development and function, alterations in BDNF levels are reported in neurological diseases^2,3^. For example, reduced BDNF levels have been detected in the brains of patients with neurodegenerative disorders such as Alzheimer’s disease^4^, Parkinson’s disease^5^, and Huntington’s disease^6^, as well as neuropsychiatric disorders such as depression^7^ and schizophrenia^8^. As well as lower BDNF levels in cerebrospinal fluid (CSF) from patients with Alzheimer’s disease, reduced CSF levels of BDNF are also associated with progression from mild cognitive impairment to Alzheimer’s disease^9^. Indeed, these findings strongly support the notion that BDNF is not only a biomarker but also a potential drug target for these diseases. In support, increased BDNF levels in the hippocampus have been found in post-mortem brains from individuals treated with antidepressant medications^10^. Further, fingolimod (a sphingoshine 1 phosphate receptor modulator) rescues reduced levels of BDNF in several regions of the brain including the striatum and hippocampus, and ameliorates symptoms in a mouse model of Rett syndrome^11^. Furukawa-Hibi *et al*., (2011) have shown that hydrophobic dipeptide leucyl-isoleucine exerts an antidepressant-like effect by inducing BDNF expression^12^. Moreover, 7,8-dihydroxyflavone, an agonist of the BDNF receptor tropomyosin receptor kinase B (TrkB) has been found to have beneficial effects in a number of neurodegenerative and neuropsychiatric disorders such as Alzheimer’s disease^13,14^, Parkinson’s disease^15^, schizophrenia^16^, and major depression^17,18^. Together, these reports strongly suggest that identifying inducers of BDNF expression, or activators of TrkB, may contribute to identifying candidate agents and improving symptoms of neurological diseases. However, there is no appropriate method that easily evaluates the ability of appropriate agents to induce BDNF expression in neurons.

Previously, we generated a novel transgenic mouse strain, termed *Bdnf-Luciferase* transgenic (*Bdnf-Luc*) mice, to measure and visualise changes in *Bdnf* expression using firefly luciferase as an imaging probe^19^. In this transgenic mouse, the firefly luciferase gene was introduced into the translation start site of the mouse *Bdnf* gene using a bacterial artificial chromosome (BAC). Accordingly, transcriptional regulation of *Luciferase* is under the control of endogenous *Bdnf* transcription. Therefore, expression of *Luciferase* reflects that of endogenous *Bdnf*. Using this approach, we successfully visualised *Bdnf* transcriptional activation by detecting bioluminescence from each cell *in vitro* by microscopic time-lapse imaging^19,20^. We also evaluated changes in *Bdnf* expression by measuring luciferase activity in a mass of cells.

Here, we generated a high-throughput screening method to screen potential inducers of neuronal *Bdnf* transcription using primary cultures of *Bdnf-Luc* mouse cortical cells in a 96-well format. Using this screening assay, we detected a series of *Bdnf* inducers from neurotransmitter and herbal libraries. We focused on the effect of an active extract prepared from Ginseng Radix (GIN), which was identified from the herbal library, and found to activate endogenous *Bdnf* expression in cultured cortical cells. Consequently, we show that this extract induces *Bdnf* expression via cAMP-response element (CRE)-binding protein (CREB)-dependent transcription. Taken together, this screening assay can identify inducers of *Bdnf* expression in neurons, and may be useful for mining candidates for therapeutic agents of dementia and other BDNF-related neurological diseases as well as inhibitors of aging-related cognitive impairment.

## Results

### Construction of a screening assay using primary cultures of *Bdnf-Luc* mouse cortical cells

**Figure 1:**
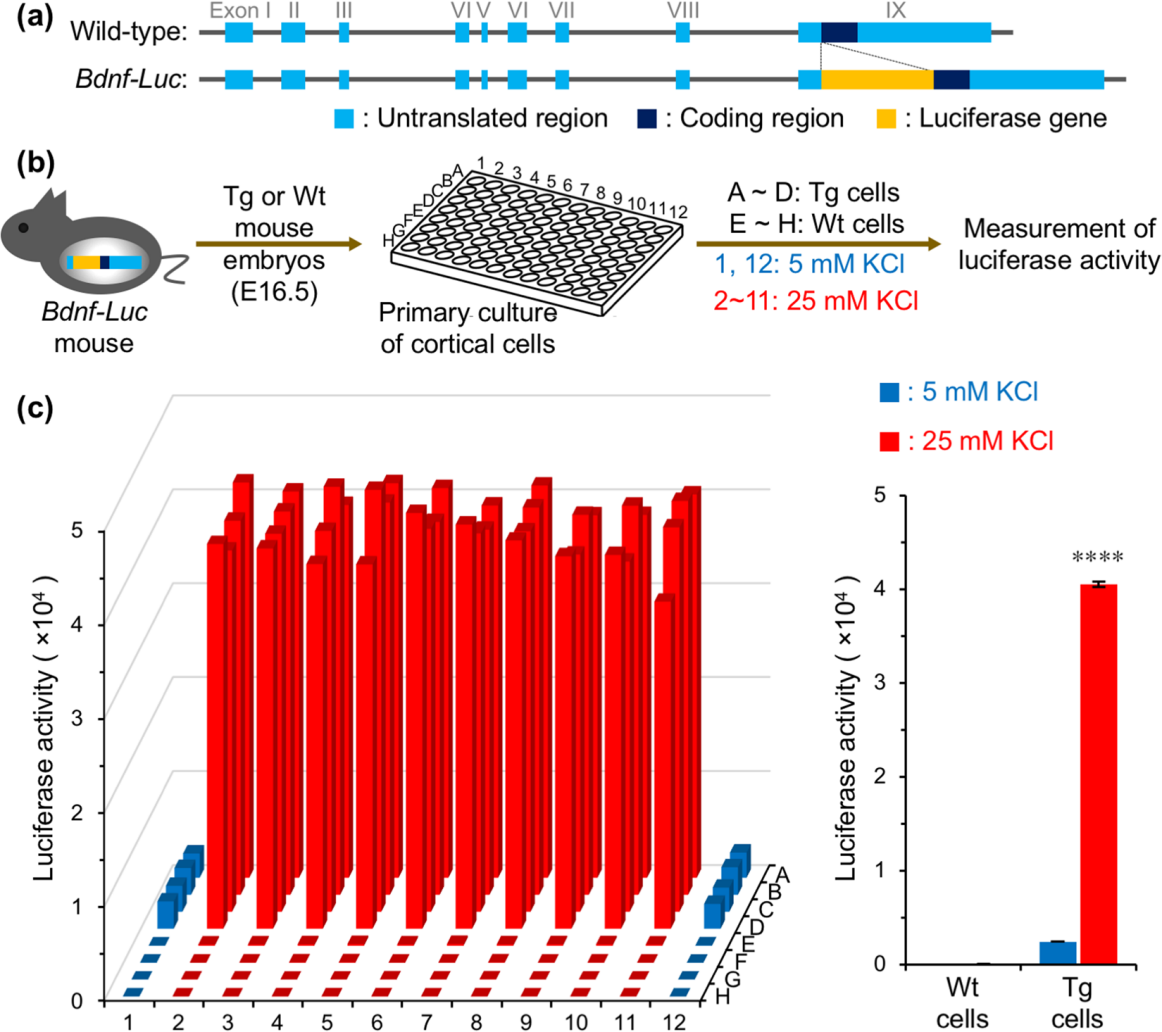
Construction of a screening assay to examine activity of *Bdnf* transcription using primary cultures of *Bdnf-Luc* mouse cortical cells in a 96-well format. (**a**) Schematic of wild-type *Bdnf* and *Bdnf-Luciferase* on a BAC. Detailed information was described previously^19,20^. (**b**) Schematic for validating the screening assay system. In 96-well culture plates, *Bdnf-Luc* mouse (Tg) cortical cells and wild-type (Wt) mouse cortical cells were seeded into wells along lines A–D and lines E–H, respectively. At 13 DIV, cells in wells of columns 2-11 were treated with a high concentration (final 25 mM) of KCl for 6 h. Cells in the wells of columns 1 and 12 were treated with PBS for 6 h. (**c**) Luciferase activity of each well (left) and the average of luciferase activity (right). Means ± SEM (n = 8 (5 mM KCl), or 40 (25 mM KCl)), ****p < 0.0001 vs. 5 mM KCl (unpaired *t*-test).

We have previously demonstrated that induction of *Bdnf* transcription could be detected by measuring luciferase activity and *in vitro* bioluminescence imaging using primary cultures of cortical cells prepared from *Bdnf-Luc* mouse embryos^19,20^. Here, we used these cultured cortical cells to screen for potential inducers of *Bdnf* transcription in neurons. We prepared primary cultures of cortical cells from *Bdnf-Luc* and wild-type mice at embryonic day 16.5 using 96-well culture plates (Fig. 1a,b). To induce membrane depolarization, cultured cells (at 13 days *in vitro* [DIV]) were treated with high concentrations (25 mM) of KCl for 6 h (Fig. 1b) (which evokes Ca^2+^ influx into neurons mainly via L-type voltage-dependent Ca^2+^ channels [L-VDCC] and subsequently activates *Bdnf* transcription^21^), then luciferase activity was measured in each well. Compared with controls (5 mM KCl), we detected higher luciferase activity in primary cultures of *Bdnf-Luc* cortical cells treated with 25 mM KCl (Fig. 1c). In contrast, we barely detected luciferase activity in primary cultures of cortical cells prepared from wild-type mouse embryos in the absence or presence of high KCl concentrations (Fig. 1c).

**Figure 2:**
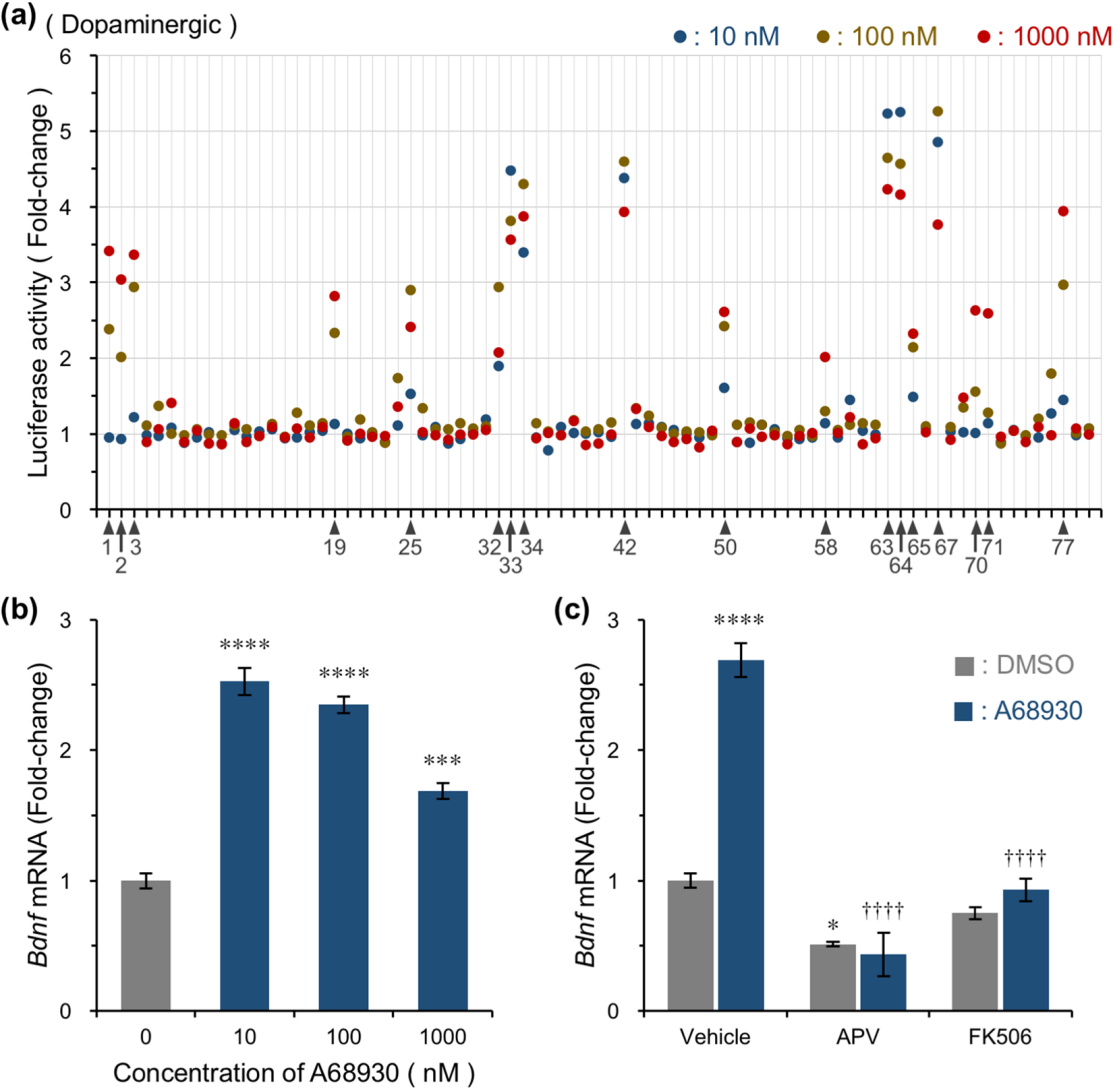
Screening activators of *Bdnf* transcription from a dopaminergic library. (**a**) Representative result obtained using a commercially available dopaminergic library. Each compound was added into *Bdnf-Luc* cortical cells at 13 DIV at a final concentration of 10, 100, or 1000 nM, and luciferase activity was measured in each well 6 h later. Arrowheads show active compounds (that increased luciferase activity by more than 2-fold). For compound names, see Supplementary Table S1. (**b**) Changes in *Bdnf* expression in the presence of dopamine D_1_ agonist A68930 in primary cultures of rat cortical cells. At 13 DIV, cells were treated with A68930 for 1 h, and then total RNA was prepared for RT-PCR analysis. Means ± SEM (n = 3), ***p < 0.001 and ****p < 0.0001 vs. 0 nM (one-way ANOVA with Dunnett’s multiple comparisons test). (**c**) Effect of APV or FK506 on A68930-induced *Bdnf* expression in cultured rat cortical cells. APV (200 μM) or FK506 (5 μM) was added 10 min before the addition of A68930 (100 nM). Means ± SEM (n = 3), *p < 0.05 and ****p < 0.0001 vs. DMSO/vehicle, ^††††^*p* < 0.0001 vs A68930/vehicle (two-way ANOVA with Tukey’s multiple comparisons test).

Next, we used commercially available neurotransmitter libraries (ENZO Life Sciences, Inc.; Supplementary Table S1) to examine the ability of each compound to induce *Bdnf* expression. Cultured cells prepared from *Bdnf-Luc* mice were treated with each test compound for 6 h, then luciferase activity was measured in each well. We compared luciferase activities of vehicle (DMSO) controls with each compound. Compounds that increased luciferase activity by more than 2-fold were defined as active. Among seven neurotransmitter libraries (dopaminergic, adrenergic, serotonergic, cholinergic, histaminergic, metabotropic glutamatergic, and GABAergic; Supplementary Table S1), active compounds were particularly detected in the dopaminergic library (Fig. 2a). We also identified several active compounds from the adrenergic library (Supplementary Fig. S1a) but rarely from other libraries (Supplementary Fig. S1b–f). In the dopaminergic library, most active compounds were classified as dopamine agonists and dopamine D_1_ agonists. Meanwhile, active compounds in the adrenergic library were agonists for the adrenaline β receptor. These results corresponded with our previous study on G_s_-coupled dopamine D_1_- and adrenaline β receptor-mediated *Bdnf* transcription via the *N*-methyl-D-aspartate receptor (NMDAR)/calcineurin/CREB-regulated transcriptional coactivator 1 (CRTC1)/CREB pathway^19^. SKF38393 and isoproterenol have been previously shown to activate *Bdnf* transcription^19^, and were included in the dopaminergic and adrenergic libraries used in this study (Fig. 1a [No. 34] and Supplementary Fig. S1a [No. 4], respectively). We chose one compound from the dopaminergic library, A68930 (a selective D_1_ agonist; Fig. 1a [No. 33]), to determine whether it induced endogenous *Bdnf* expression in neurons. Using primary cultures of rat cortical cells, we found that endogenous *Bdnf* mRNA expression levels were increased by A68930 (Fig. 2b). Corresponding with a previous finding that dopamine D_1_ receptor activation induced *Bdnf* transcription via the NMDAR/calcineurin pathway^19^, A68930-induced *Bdnf* transcription was completely blocked by the NMDAR antagonist D-2-amino-5-phosphonovaleric acid (APV) and calcineurin inhibitor FK506 (Fig. 2c). We also chose several compounds, propylnorapomorphine (Fig. 1a [No. 77]), cabergoline (Fig. 1a [No. 50]), and norepinephrine (Supplementary Fig. S1a [No. 78]), from these libraries, and found that they increased endogenous *Bdnf* mRNA expression levels (Supplementary Fig. S2a–c). In contrast, inactive serotonin (Supplementary Table S1 [Serotonergic library, No. 1] and Supplementary Fig. S1b), only slightly affected mRNA levels (Supplementary Fig. S2d). Focusing on these compounds, we found that changes in endogenous *Bdnf* mRNA expression levels were strongly correlated with those in luciferase activity (Supplementary Fig. S2e, r = 0.931, *p* < 0.0001). Furthermore, we identified three active compounds, all of which were GABA_A_ receptor antagonists, from the GABAergic library (Supplementary Fig. S1f [No. 29, 34, and 35]). This corresponds with a previous result showing that a GABA_A_ receptor antagonist blocked inhibitory neurotransmission and evoked neuronal excitation-induced *Bdnf* transcription in mature neurons^22^. Taken together, we showed that compounds activating *Bdnf* transcription in neuronal cells can be screened using primary cultures of *Bdnf-Luc* cortical cells in a 96-well format.

### Identification of herbal extracts that induce *Bdnf* expression

**Figure 3:**
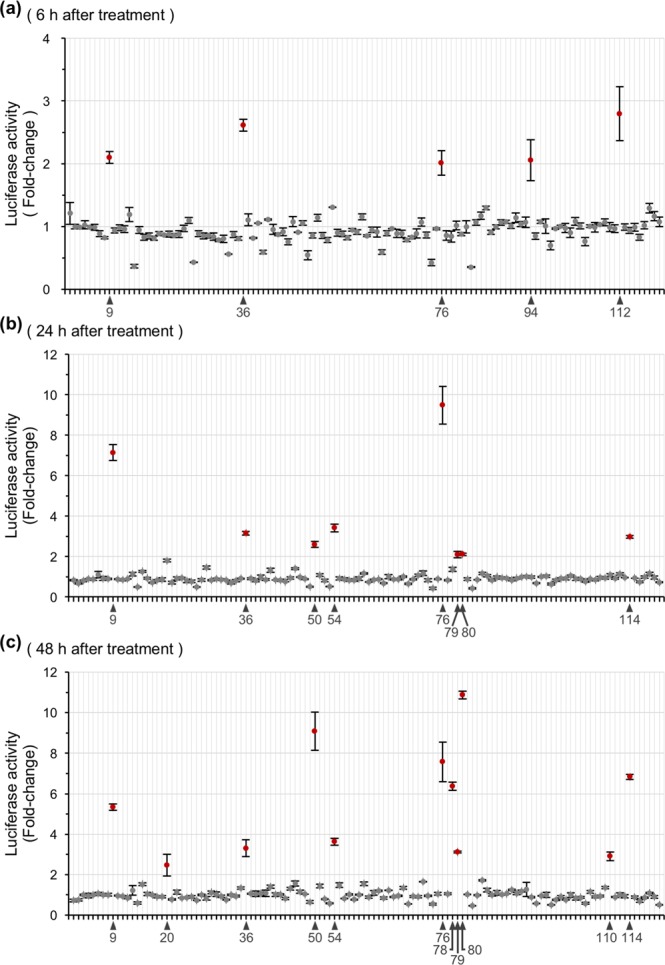
Screening activators of *Bdnf* transcription from an herbal extract library. Representative results obtained using an herbal extract library consisting of 120 herbal extracts. Each extract was added into *Bdnf-Luc* cortical cells at 13 DIV at a final concentration of 500 μg/mL. Luciferase activity was measured in each well at 6 h (**a**), 24 h (**b**), or 48 h (**c**) after addition of extract. Means ± SEM (n = 3). Active extracts (that increased *Bdnf* induction by more than 2-fold) are shown in red.

**Figure 4:**
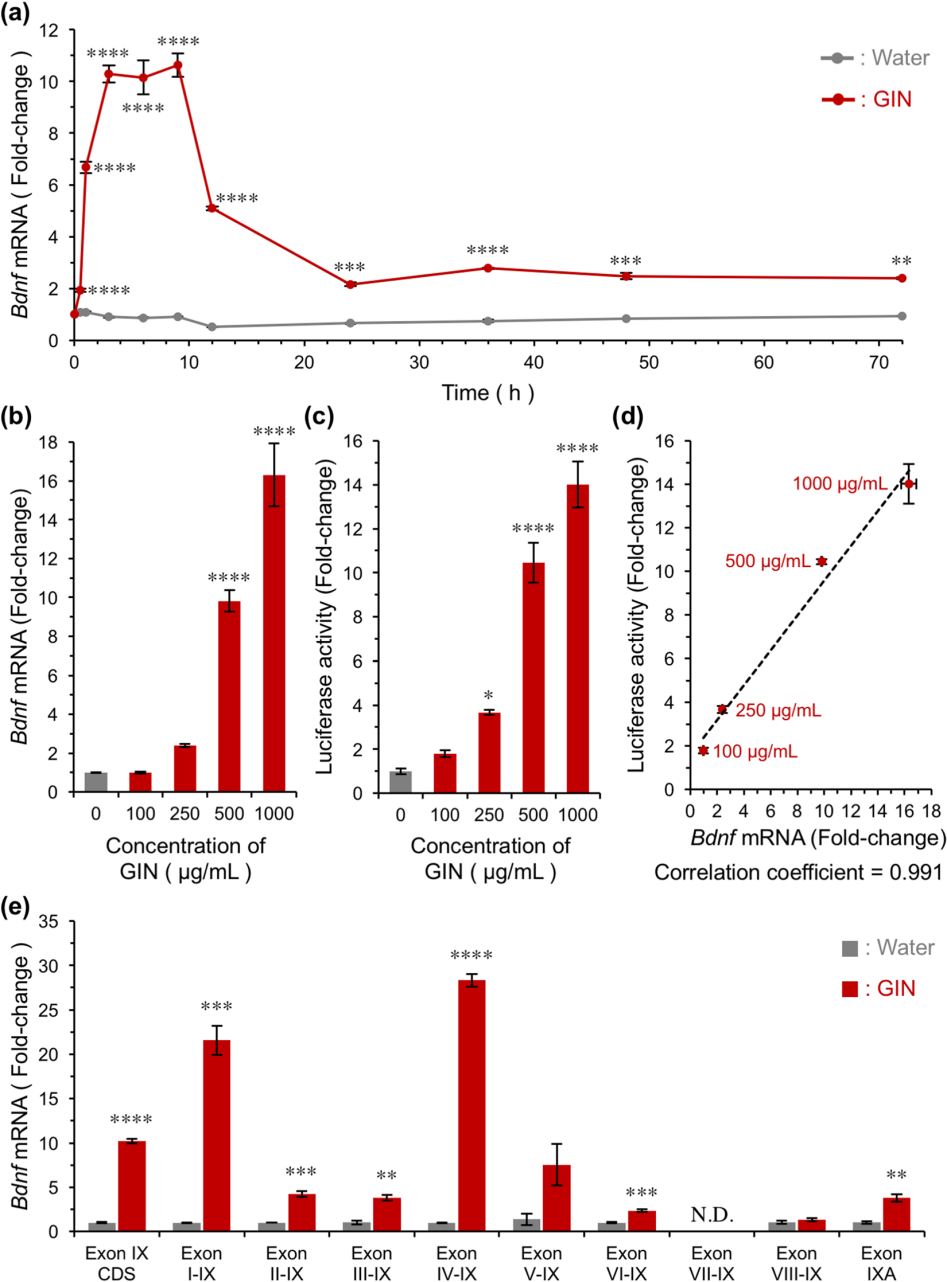
GIN induces endogenous *Bdnf* expression in primary cultures of rat cortical cells. (**a**,**b**) Time-course (**a**) and dose-dependency (**b**) of changes in *Bdnf* mRNA in the presence of GIN in primary cultures of rat cortical cells. (**a**) Cells were treated with 500 μg/mL GIN and total RNA was prepared at the indicated time. Means ± SEM (n = 3), **p < 0.01, ***p < 0.001, and ****p < 0.0001 vs. water at the same time point (two-way ANOVA with Tukey’s multiple comparisons test). (**b**) Cells were treated with different concentrations of GIN, and total RNA was prepared 3 h after treatment. Means ± SEM (n = 3), ****p < 0.0001 vs. 0 μg/mL (one-way ANOVA with Dunnett’s multiple comparisons test). (**c**) *Bdnf-Luc* cortical cells were seeded into a 96-well culture plate and cultured for 13 days. Cells were then treated with different concentrations of GIN, and luciferase activity in each well was measured 6 h after treatment. Means ± SEM (n = 3), *p < 0.05 and ****p < 0.0001 vs. 0 μg/mL (one-way ANOVA with Dunnett’s multiple comparisons test). (**d**) Relationship between changes in endogenous *Bdnf* mRNA expression levels (**b**) and those in luciferase activity (**c**) was analysed using a correlation coefficient test. Statistical analysis was performed by Pearson’s correlation coefficient test. (**e**) The effect of GIN on the expression of 5′ exon-specific *Bdnf* mRNA in cultured rat cortical cells. Cells were treated with 500 μg/mL GIN and total RNA prepared 3 h after the treatment. Means ± SEM (n = 3), **p < 0.01, ***p < 0.001, and ****p < 0.0001 vs. water (unpaired *t*-test). N.D.: not detected.

Next, we determined whether the current screening assay could be used to identify active crude extracts. We determined the ability of a series of extracts prepared from herbal medicines to induce *Bdnf* transcription in neurons. We used a library consisting of 120 herbal extracts (Supplementary Table S2). Cultured *Bdnf-Luc* cortical cells were treated with herbal extracts at a final concentration of 500 μg/mL for 6 h (Fig. 3a), 24 h (Fig. 3b), or 48 h (Fig. 3c), then luciferase activity was measured in each well. We found that five active herbal extracts (Ginseng Radix [Nos. 9 and 36], Zanthoxyli Piperiti Pericarpium [No. 76], Aconiti Radix Processa [No. 94], and Sinomeni Caulis et Rhizoma [No. 112]) increased luciferase activity at 6 h after their addition (Fig. 3a). The number of active extracts increased when cells were treated with each extract for 24 or 48 h. We found eight herbal extracts (Ginseng Radix [Nos. 9 and 36], Polygoni Multiflori Radix [No. 50], Panacis Notoginseng Radix [No. 54], Zanthoxyli Piperiti Pericarpium [No. 76], Rhei Rhizoma [No. 79], Uncariae Uncis Cum Ramulus [No. 80], and Alpiniae Officinari Rhizoma [No. 114]) after 24 h treatment (Fig. 3b), and 11 herbal extracts (Ginseng Radix [Nos. 9 and 36], Amomi Semen [No. 20], Polygoni Multiflori Radix [No. 50], Panacis Notoginseng Radix [No. 54], Zanthoxyli Piperiti Pericarpium [No. 76], Paeoniae Radix Rubra [No. 78], Rhei Rhizoma [No. 79], Uncariae Uncis Cum Ramulus [No. 80], Ephedrae Herba [No. 110], and Alpiniae Officinari Rhizoma [No. 114]) after 48 h treatment (Fig. 3c). We chose a subset of active extracts and confirmed that they significantly increased the endogenous expression of *Bdnf* mRNA in cultured rat cortical cells (Fig. 4a, Ginseng Radix [No. 36]; and Supplementary Fig. S3a, Zanthoxyli Piperiti Pericarpium [No. 76]; S3b, Ginseng Radix [No. 9]; S3c, Polygoni Multiflori Radix [No. 50]; S3d, Panacis Notoginseng Radix [No. 54]; S3e, Rhei Rhizoma [No. 79]; S3f, Ephedrae Herba [No. 110]; S3g, Alpiniae Officinari Rhizoma [No. 114]; S3h, Sinomeni Caulis et Rhizoma [No. 112]; S3i, Uncariae Uncis Cum Ramulus [No. 80]). We particularly focused on the effect of the extract prepared from Ginseng Radix (GIN), and found that GIN increased levels of endogenous *Bdnf* mRNA (Fig. 4b), as well as luciferase activity (Fig. 4c), in a dose-dependent manner. Changes in endogenous mRNA levels were strongly correlated with those in luciferase activity (Fig. 4d, *r* = 0.991, *p* < 0.01). We also examined the effect of GIN on levels of exon-specific *Bdnf* mRNA, and found higher induction levels of exon I and exon IV-containing *Bdnf* mRNA (Fig. 4e), both of which are major components of activity-regulated *Bdnf* transcription in neurons^23,24^.

As shown in Fig. 3, several extracts reduced luciferase activity. We demonstrated that expression levels of *Luciferase* and endogenous *Bdnf* mRNA were reduced after addition of the transcription inhibitor actinomycin D (Supplementary Fig. S4a, t_1/2_ = 5.21 h [*Bdnf*], 3.67 h [*Luciferase*]), in cultured *Bdnf-Luc* cortical cells. In contrast, luciferase activity did not significantly alter after actinomycin D treatment (Supplementary Fig. S4b), suggesting that luciferase protein was stably expressed in the cells. Therefore, reduced luciferase activity by these extracts was not caused by the repression of *Bdnf* transcription.

We found that some compounds and extracts increased luciferase activity (Figs 2a and 3, Supplementary Fig. 1). To exclude the possibility that increased luciferase activity reflected increased stability of luciferase protein and/or the enhancement of luciferase translation, we used actinomycin D. We found that high concentrations (25 mM) of KCl, norepinephrine, and GIN significantly increased luciferase activity using a screening assay (Supplementary Fig. S4c), which agreed with our earlier results (Figs 1c and 3a, Supplementary Fig. S1a). However, these increases were not observed in the presence of actinomycin D (Supplementary Fig. S4c), suggesting that increased luciferase activity mainly reflected the activation of *Bdnf* transcription.

### Ginsenosides or gintonin do not participate in activation of *Bdnf* transcription

**Figure 5:**
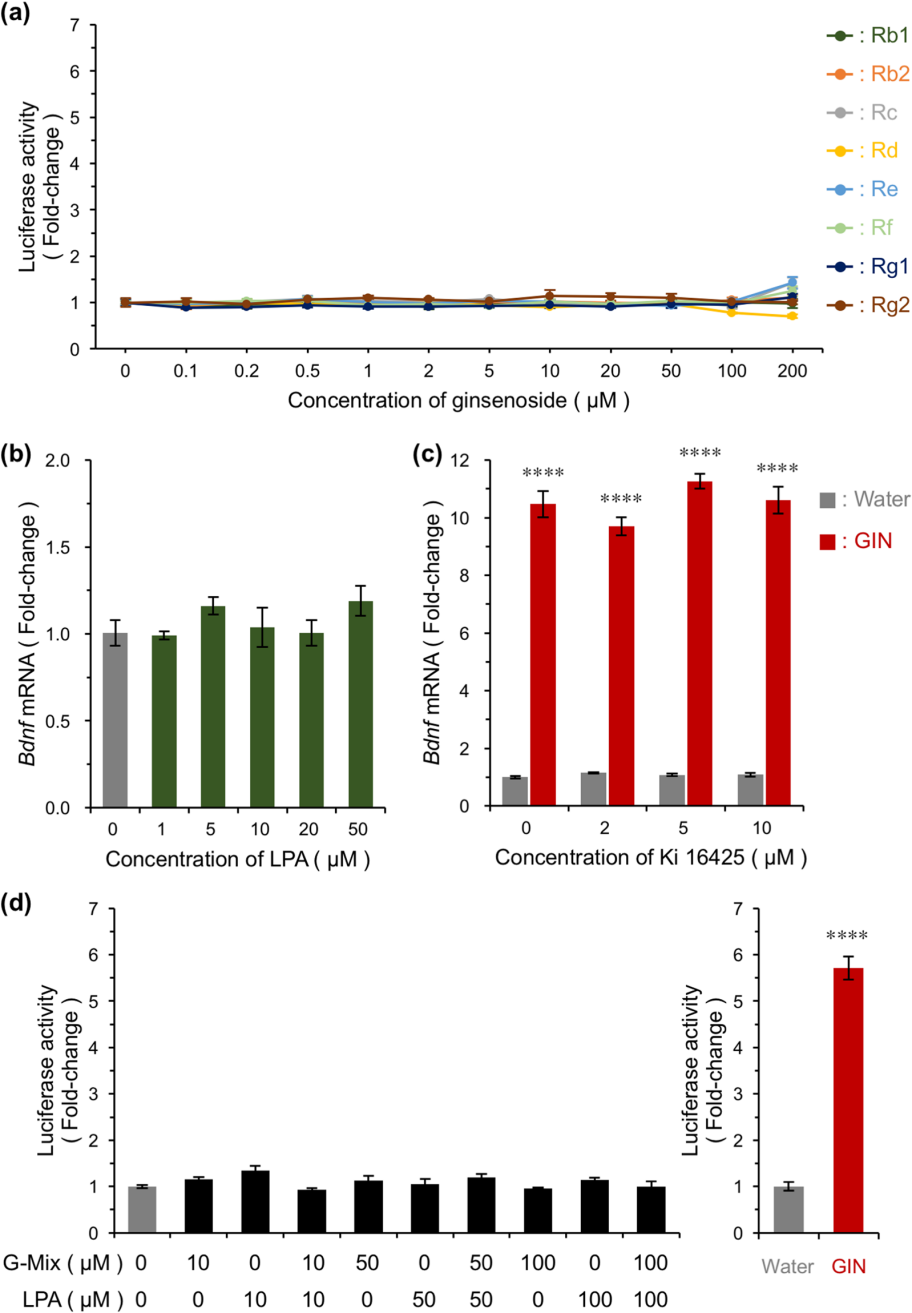
No contribution of ginsenosides or LPA receptors to GIN-induced *Bdnf* expression. (**a**) Representative result obtained using a series of ginsenosides consisting of eight types: Rb1, Rb2, Rc, Rd, Re, Rf, Rg1, and Rg2. Each ginsenoside was added into *Bdnf-Luc* cortical cells at 13 DIV at the indicated concentrations, and luciferase activity was measured in each well 6 h after addition. Means ± SEM (n = 3). (**b**) Effect of the LPA_1/2_ receptor agonist, oleoyl-L-α-lysophosphatidic acid (LPA), on *Bdnf* expression in primary cultures of rat cortical cells. Cells were treated with LPA at the indicated concentrations, and total RNA was prepared 3 h after treatment. Means ± SEM (n = 3). (**c**) Effect of the LPA_1/3_ receptor antagonist Ki 16425 on the GIN-induced *Bdnf* expression. Ki 16425 was added to cells at the indicated concentration 10 min before the addition of GIN (500 μg/mL). Total RNA was prepared 3 h after treatment to examine changes in *Bdnf* expression by RT-PCR. Means ± SEM (n = 3), ****p < 0.0001 vs. water in the absence or presence of Ki 16425, (two-way ANOVA with Tukey’s multiple comparisons test). (**d**) Effect of a combination of ginsenosides and/or LPA on luciferase activity in cultured *Bdnf-Luc* cortical cells (left). Major ginsenosides (ginsenoside Rb1, Rc, Rd, Re, and Rg1) in GIN (Supplementary Table S2, No. 36 (refer to database URL)) were mixed (final concentrations of each ginsenoside; 10, 50, or 100 μM). Mixed ginsenosides (G-Mix) was added into *Bdnf-Luc* cortical cells at 13 DIV with or without LPA (final concentration; 0, 10, 50, or 100 μM). GIN was used as a positive control (right). Luciferase activity in each well was measured 6 h after addition. Means ± SEM (n = 6–8). ****p < 0.0001 vs. water (unpaired *t*-test).

Next, we sought to identify active compounds from GIN. Ginsenosides are well-known active compounds of Ginseng Radix^25^. Here, we examined eight types of ginsenosides (specifically, ginsenoside Rb1, Rb2, Rc, Rd, Re, Rf, Rg1, and Rg2), and determined the activity of these compounds using a screening assay. Cultured *Bdnf-Luc* cortical cells were treated with each ginsenoside for 6 h, then luciferase activity was measured in each well. Although GIN increased luciferase activity in a dose-dependent manner (Fig. 4c), no individual ginsenoside significantly increased luciferase activity (Fig. 5a), suggesting that individual ginsenosides do not affect *Bdnf* expression. In contrast, gintonin, a lysophosphatidic acids (LPA)-protein complex mainly containing LPA C_18:2_, has previously been isolated as an active component of Ginseng Radix^26^. LPA receptors are G protein-coupled receptors (GPCR) such as G_i/o_, G_12/13_, G_q/11_, and G_s_-coupled receptors. We previously reported that stimulation of G_s_- and G_q_-coupled receptors activates *Bdnf* transcription via a NMDAR/Ca^2+^/calcineurin/CRTC1/CREB-dependent pathway^19^, suggesting that GIN-regulated *Bdnf* expression may be regulated by gintonin-regulated activation of G_q/11_ and/or G_s_-coupled LPA receptors. However, the LPA_1/2_ agonist oleoyl-L-α-lysophosphatidic acid did not affect *Bdnf* mRNA expression (Fig. 5b). Additionally, the LPA_1/3_ antagonist, Ki 16425, did not suppress GIN-induced *Bdnf* expression (Fig. 5c). We also examined whether a series of ginsenosides with or without LPA_1/2_ agonist could affect *Bdnf* expression using a screening assay. We chose major ginsenosides, ginsenoside Rb1, Rc, Rd, Re, and Rg1, in an extract of Ginseng Radix (Supplementary Table S2, No. 36, refer to database URL). These ginsenosides and/or LPA_1/2_ agonist at different concentrations were added to cultured *Bdnf-Luc* cortical cells for 6 h, then luciferase activity was measured. As shown in Fig. 5d, luciferase activity was not affected by a combination of ginsenosides with or without oleoyl-L-α-lysophosphatidic acid, whereas it was significantly increased by GIN. We also prepared a methanol eluate fraction with enriched ginsenosides^27^ from a water extract of Ginseng Radix, using a Diaion HP-20 column chromatography (Supplementary Fig. S5a,b). A screening assay showed that luciferase activity was unaffected by this methanol eluate fraction but was increased by water extract (Supplementary Fig. S5c), supporting our finding of a lower effect of individual ginsenoside on luciferase activity (Fig. 5a). Taken together, GIN was shown to activate endogenous *Bdnf* expression in neurons, yet individual ginsenosides and/or activation of LPA receptors by gintonin does not participate in its induction.

We also used a compound library consisting of 96 types of herbal medicine-derived compounds (Supplementary Table S3), and examined the ability of each compound to induce *Bdnf* expression in primary cultures of *Bdnf-Luc* cortical cells. Cultured *Bdnf-Luc* cortical cells were treated with each compound for 6 h, then luciferase activity was measured in each well. We found that six compounds increased luciferase activity (Supplementary Fig. S6). Among these active compounds, aconitine (No. 1), hypaconitine (No. 56), and mesaconitine (No. 81) are components of Aconiti Radix Processa, the extract of that was identified as active using our screening assay (Fig. 3a [No. 94]). Aconitum alkaloids reportedly bind the α subunit of voltage-dependent Na^+^ channels and inhibit their inactivation, resulting in the induction of membrane depolarization in neurons^28^. Considering a previous report showing activity-dependent *Bdnf* transcription in neurons^21^, this suggests that the extract prepared from Aconiti Radix Processa may induce activity-dependent *Bdnf* expression mediated by Aconitum alkaloids.

### Intracellular signalling pathways contributing to GIN-induced *Bdnf* transcription

**Figure 6:**
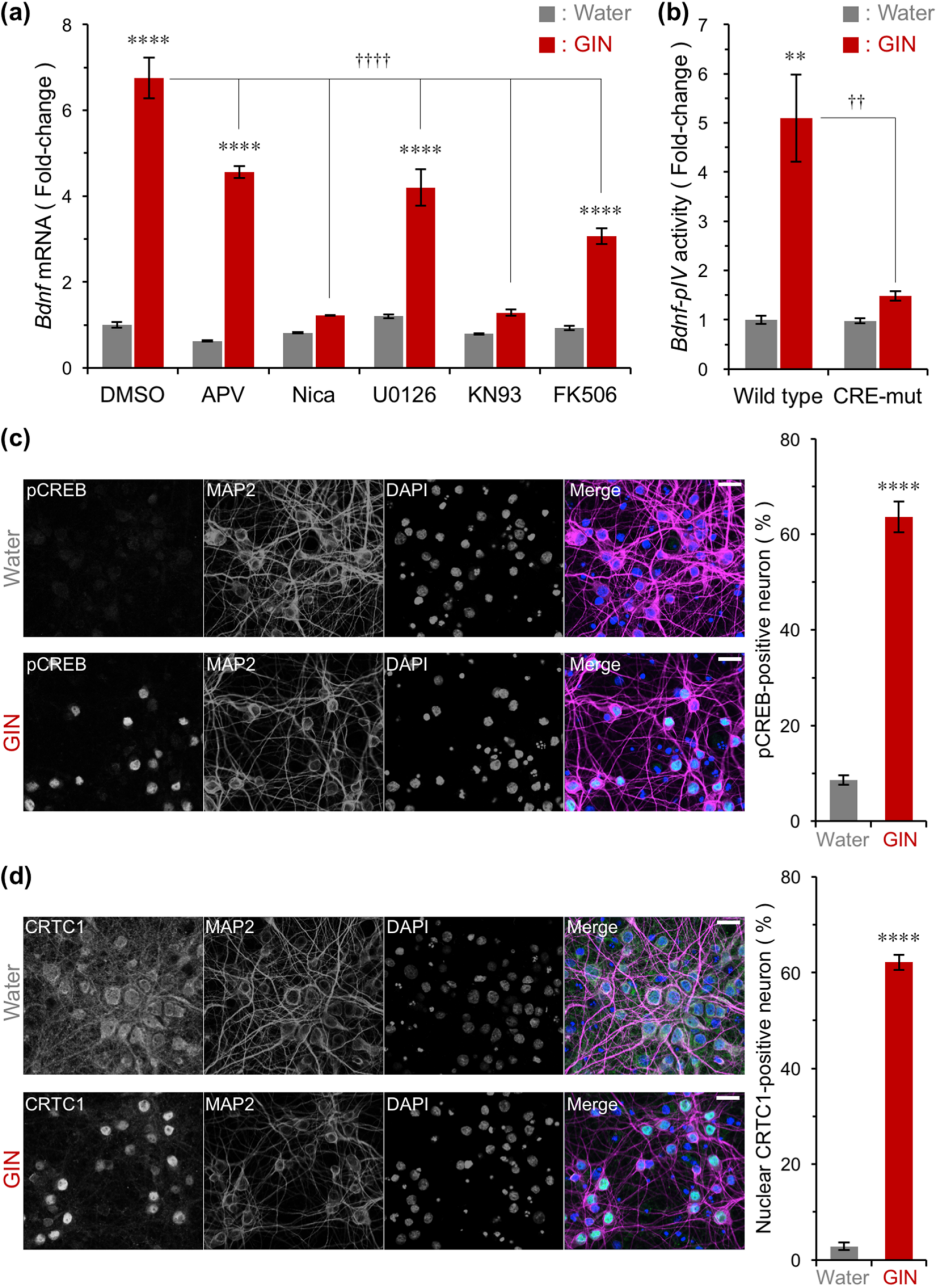
GIN-induced *Bdnf* transcription via CREB-dependent pathways. (**a**) Effects of blocking Ca^2+^ entry sites and Ca^2+^ signalling inhibitors on GIN-induced *Bdnf* expression in primary cultures of rat cortical cells. APV (200 μM), nicardipine (Nica, 5 μM), U0126 (20 μM), KN93 (10 μM), or FK506 (5 μM) were added 10 min before the addition of GIN (500 μg/mL). Total RNA was prepared 3 h after treatment to examine changes in *Bdnf* expression by RT-PCR. Means ± SEM (n = 3), ****p < 0.0001 vs. water, ^††††^*p* < 0.0001 vs. DMSO/GIN (two-way ANOVA with Tukey’s multiple comparisons test). (**b**) Changes in *Bdnf* promoter IV (*Bdnf-pIV*) activity in the presence of GIN. Transfected cortical cells were treated with 500 μg/mL GIN for 6 h, and luciferase activity measured. Means ± SEM (n = 3), **p < 0.01 vs. Wild type/water, ^††^*p* < 0.01 vs. Wild type/GIN (two-way ANOVA with Tukey’s multiple comparisons test). (**c**,**d**) (left) Representative images of phosphorylated CREB at serine 133rd (**c**) or subcellular localization of CRTC1 (**d**), and (right) percentage of phospho-CREB-positive (**c**) or nuclear CRTC1-positive (**d**) neurons. Cultured rat cortical cells were treated with 500 μg/mL GIN for 30 min, then cells were fixed for immunostaining. Scale bar = 20 μm. Means ± SEM (n = 3), ****p < 0.0001 vs. water (unpaired *t*-test).

To identify the signalling pathways involved in GIN-induced *Bdnf* expression, we investigated the involvement of Ca^2+^ signalling pathways, which are core pathways of *Bdnf* regulation in neurons^19^. Nicardipine, a L-VDCC blocker, and KN93, a Ca^2+^/calmodulin-dependent protein kinase (CaMK) inhibitor, almost completely blocked GIN extract-induced *Bdnf* expression (Fig. 6a). Further, APV partially blocked the induction (Fig. 6a). The induction of *Bdnf* mRNA under GIN treatment was partially prevented by FK506 or mitogen-activated protein kinase/extracellular signal-regulated protein kinase kinase 1/2 (MEK1/2) inhibitor U0126, respectively (Fig. 6a).

Using a luciferase-based promoter assay, we found that GIN activated *Bdnf* promoter IV (*Bdnf-pIV*; Fig. 6b), which is a core promoter involved in activity-regulated *Bdnf* transcription^21,29^. Furthermore, activation of the promoter was barely observed when CRE (also known as CaRE3^21,29^) on *Bdnf-pIV* was mutated (Fig. 6b). Taken together with the results obtained from a series of inhibitors (Fig. 6a), CREB-mediated transcription evoked mainly by L-VDCC/Ca^2+^/CaMKs appears to be involved in the activation of *Bdnf* transcription by GIN.

We next examined whether CREB-dependent transcription was enhanced by GIN. It is well known that CREB phosphorylation at serine 133rd and nuclear translocation of the CREB coactivator CRTC1 contributes to the activation of CREB-dependent transcription by neuronal activity^30^. Here, we found that GIN increased the number of neurons with phosphorylated CREB at serine 133rd and nuclear CRTC1 (Fig. 6c,d). These results indicate that GIN activates *Bdnf* transcription via Ca^2+^ signal-mediated CREB-dependent transcription.

We also comprehensively analysed gene expression profiles regulated by GIN in cultured rat cortical cells. We chose transcripts with fold-change values of greater than or less than 2 (upregulated and downregulated, respectively). We found that 63 and eight transcripts were significantly upregulated and downregulated, respectively, by treatment of the cells with GIN for 3 h (Supplementary Table S4). We also found that GIN increased the expression of *Bdnf* and other genes encoding plasticity-related factors such as *Nr4a1*, *Nr4a2*, and *Homer1* (Supplementary Table S4a)^31^.

Finally, we examined whether luciferase activity would be measured in other cell types. It has been reported that membrane depolarization induces Ca^2+^ influx via L-VDCCs and subsequently increases *Bdnf* expression in primary cultures of cerebellar granule cells^32,33^. Here, we prepared primary cultures of cerebellar granule cells from *Bdnf-Luc* mice brain using a 96-well culture plate, then cultured cells at 7 DIV were treated with high concentration (25 mM) of KCl for 6 h. We found that luciferase activity was significantly increased by KCl, and the increases were completely abolished in the presence of nicardipine (Supplementary Fig. S7). Thus, it is strongly suggested that inducers of *Bdnf* expression could be identified in other cell types.

## Discussion

Here, we developed a screening assay to identify inducers of *Bdnf* transcription using primary cultures of cortical cells prepared from *Bdnf-Luc* mouse embryos. In this transgenic mouse line, the firefly luciferase gene has been introduced into the translation start site of *Bdnf* using a BAC clone containing the entire mouse *Bdnf* gene. Therefore, in contrast to the construct in which a truncated promoter region is fused to a reporter gene, luciferase expression from BAC-based *Bdnf-Luc* is predicted to reflect endogenous *Bdnf* expression. In fact, our previous study showed that changes in *Bdnf* expression could be successfully evaluated by measuring luciferase activity as well as a bioluminescence signal in cultured cortical cells^19,20^. In this study, we successfully detected membrane depolarization-induced increases in luciferase activity, with high signal/noise ratios, using primary cultures of *Bdnf-Luc* cortical cells in 96 well-format culture plates. Using this method, we identified compounds and herbal extracts from a series of libraries that activated *Bdnf* transcription in neurons.

We defined compounds that increased luciferase activity by more than 2-fold as active ones, and presume that this threshold is necessary to identify inducers of *Bdnf* mRNA expression levels in neurons. For example, extracts prepared from Ginseng Radix (No. 9, fold-induction value = 2.10) and Zanthoxyli Piperiti Pericarpium (No. 76, fold-induction value = 2.01) increased endogenous *Bdnf* mRNA expression levels. In contrast, serotonin, which did not increase luciferase activity (fold-induction value = 1.29 [10 nM], 1.13 [100 nM], 1.32 [1000 nM], respectively), only slightly affected the mRNA levels at any concentrations. However, this threshold was not sufficient for identifying *Bdnf* inducers. We found that 100 nM propylnorapomorphine and 100 nM cabergoline increased luciferase activity (fold-induction value; 100 nM propylnorapomorphine = 2.96, 100 nM cabergoline = 2.42, respectively), yet these compounds at the same concentration did not significantly affect endogenous *Bdnf* mRNA expression levels. This might reflect a time lag between changes in endogenous *Bdnf* transcription and those in luciferase activity. For instance, it has been reported that activation of *Bdnf* transcription peaked at 1 h after depolarization^21^ whereas luciferase activity increased gradually and peaked at approximately 7 h after depolarization^20^. In support of this, time-course changes in luciferase activity after the addition of herbal extracts did not always correspond with those in endogenous *Bdnf* mRNA expression levels in our study. We suggest that this time lag should be further investigated and that endogenous *Bdnf* expression levels should be examined to determine if they are increased by active agents obtained in the current screening assay.

The most important aspect of our screening assay is that activation of *Bdnf* transcription could be evaluated in a primary neuronal culture. Previously, Jaanson *et al*., (2014) developed a similar screening assay in stable HeLa cell^34^. They also used a BAC clone and replaced the coding region of the *BDNF* with the *Renilla* luciferase-EGFP gene. Accordingly, changes in reporter gene expression were predicted to synchronise with those of endogenous *Bdnf* expression. However, this strategy enabled screening for regulators of *Bdnf* expression in HeLa cells, but not in neuronal cells, yet it is questionable whether active compounds identified in HeLa cells have the ability to induce *Bdnf* expression in neuronal cells. Ishimoto *et al*., (2012) also developed a novel screening assay to identify CREB activators in HEK293T cell lines^35^. They also used the same herbal extract library that we used in our study. However, none of the extracts that activated CREB in HEK293T cells could activate *Bdnf* expression in our current screening assay. Moreover, although the addition of GIN increased CREB phosphorylation in cultured cortical cells, GIN was not observed to activate CREB in the screening assay using HEK293T cells^35^. These results also suggest that intracellular signalling pathways and cellular responses differ between primary neuronal cells and established cell lines. Alternatively, using our current method, we can screen for inducers of *Bdnf* expression in neuronal cells and other cell types if primary cultures are prepared from tissues of interest. In support of this, we prepared primary cultures of cerebellar granule cells from *Bdnf-Luc* mice using a 96-well culture plate, and found that membrane depolarization increased luciferase activity, which agreed with previous reports regarding the activity-dependent *Bdnf* expression in cultured cerebellar granule cells^32,33^.

We found that expression levels of *Luciferase* and endogenous *Bdnf* mRNA were similarly reduced after the addition of actinomycin D. In contrast, luciferase activity did not significantly alter under the same conditions, suggesting that luciferase protein is stably expressed even if transcription is inhibited. Consequently, it would be difficult to identify negative regulators of *Bdnf* transcription using our screening assay, because luciferase protein would be stably expressed and luciferase activity would not be easily reduced even if *Bdnf* transcription is prevented by negative regulators. Nevertheless, our screening assay allows us to identify inducers of *Bdnf* transcription in neuronal cells. It is also possible that increases in luciferase activity reflect stabilization of luciferase protein and/or enhanced luciferase translation. However, we confirmed that increases in luciferase activity by depolarization, norepinephrine, and GIN were not observed in the presence of actinomycin D, suggesting a contribution of *Bdnf* transcriptional activation to increases in luciferase activity.

Although our screening assay allows us to identify *Bdnf* inducers in neurons, it is difficult to clarify how active agents increase *Bdnf* transcription. In rodents, *Bdnf* transcription is controlled by nine distinct promoters reported to be regulated by multiple transcription factors that respond to a series of intracellular signalling pathways^36,37^. Thus, a number of targets exist upstream of *Bdnf* transcription, although they may not be specific for regulating *Bdnf* expression. Therefore, it is difficult to construct a screening assay to identify active agents that specifically activate the machinery of *Bdnf* expression. However, our current assay will be useful for screening agents that are pharmacologically unvalidated compounds or crude extracts, even though the mechanisms of action of these agents on the induction of *Bdnf* transcription are unclear. In contrast to our screening assay, screenings using genetically engineered biosensors enable the identification of agents that affect specific molecules such as GPCRs. For example, an allosteric biosensor of cAMP has been developed that monitors changes in intracellular signalling evoked by the activation of G_s_-coupled GPCRs. Using HEK293 cells expressing this allosteric biosensor, Vedel *et al*., (2015) showed that β_2_ adrenergic receptor agonists including salbutamol and metaproterenol increased intracellular cAMP levels in a dose-dependent manner^38^. However, among β_2_ adrenergic agonists in the adrenergic library used in this study, only metaproterenol increased luciferase activity. Screening assays focusing on specific targets are useful for identifying active agents with certain mechanisms of action. However, active agents obtained using these methods are not always active for the purpose of identifying *Bdnf* inducers. Thus, our screening assay is beneficial for the screening of *Bdnf* inducers in neuronal cells, particularly for identifying unvalidated agents.

Our screening assay identified several herbal extracts that induced *Bdnf* transcription, and we subsequently confirmed that these extracts induced endogenous *Bdnf* expression in cultured cortical cells. Although it was difficult to identify negative regulators of *Bdnf* transcription using our screening assay, we nevertheless identified several extracts that reduced luciferase activity. One possible reason for this is reduction of cell viability based on our use of crude water extracts of herbal medicines, which might include cytotoxic components. Because the luciferase-luciferin reaction that produces bioluminescence is dependent on ATP, a reduction of cell viability would result in lower luciferase activity. In any case, we chose GIN from the active extracts, and demonstrated that it regulates *Bdnf* transcription via Ca^2+^ signal-mediated CREB-dependent transcription.

An important point that remains unclear in this study is how GIN can upregulate *Bdnf* expression in neuronal cells. We attempted to identify active compounds contributing to GIN-induced *Bdnf* transcription by examining the effect of a series of ginsenosides on *Bdnf* transcription, yet found that no ginsenoside affected transcription. There are at least two possibilities to explain this: first, multiple ginsenosides may cooperatively participate in the induction of *Bdnf* expression; or second, other components may contribute to *Bdnf* induction. We found that a mix of major ginsenosides in GIN did not affect luciferase activity. Moreover, the MeOH eluate fraction of Ginseng Radix with enriched ginsenosides also had no effect on luciferase activity, suggesting that multiple ginsenosides are less likely to affect *Bdnf* induction. Gintonin, an LPA-protein complex that acts on LPA receptors^26^, is a candidate for an active component. However, our present results showed that an LPA_1/2_ agonist had no effect on *Bdnf* mRNA expression, nor did an LPA_1/3_ antagonist on GIN-induced *Bdnf* expression. In this study, we did not examine whether other LPA receptors (G_s_-, G_q_-, G_i_-, or G_12/13_-coupled GPCRs^39^) such as LPA_4/5_ contribute to GIN-induced *Bdnf* expression. Because our current results also show a major contribution of L-VDCC/Ca^2+^ and CaMK pathways to GIN-induced *Bdnf* expression, it is unlikely that G_s_- and/or G_q_-coupled LPA receptor-mediated *Bdnf* expression is not involved in the induction. We have previously reported that G_s_- or G_q_-coupled GPCR-mediated *Bdnf* induction is mainly dependent on the NMDAR/Ca^2+^/calcineurin/CRTC1/CREB pathway^19^. If GIN-induced *Bdnf* expression is caused by the activation of G_s_- and/or G_q_-coupled LPA receptors by gintonin, GIN-induced *Bdnf* expression should be strongly inhibited by NMDAR antagonist or calcineurin inhibitor. We also confirmed that a series of ginsenosides with LPA did not affect *Bdnf* expression using a screening assay.

Because of known membrane depolarization-induced *Bdnf* expression in neurons^21^, we examined K^+^ concentrations in GIN solution but detected negligible final concentration of K^+^ in the culture medium (approximately 4 mM K^+^ in 10 mg/mL GIN solution). We previously reported that high concentrations of KCl-evoked membrane depolarization affected the expression of a large number of transcripts in cultured rat cortical cells^40^. In contrast, our microarray analysis showed that GIN likely regulates a limited number of transcripts in neurons. Further investigations are therefore necessary to reveal the cellular and molecular basis underlying GIN-induced *Bdnf* expression in neuronal cells. It is plausible that GIN exerts its neurotrophic effects via the induction of *Bdnf* expression in neurons. In support of this, GIN showed beneficial effects on neurological diseases including Alzheimer’s disease^41^ and depression^42,43^. Further work should examine whether these effects involve BDNF induction. Previous work demonstrated that the administration of ginsenosides such as Rg1 exhibited an antidepressant-like effect in mice^44^ and ameliorates memory impairment in Alzheimer’s disease model mice^45^. Although these reports show that ginsenoside rescued the reduced expression of BDNF in model animals, it is unclear whether it does so under normal conditions. Our current study is the first to show that GIN, but not active components of GIN such as ginsenoside and gintonin, directly activates *Bdnf* transcription in neurons. Thus, GIN is expected to have beneficial effects on cognitive and other neuropsychiatric functions in healthy groups as well as those affected by neurological diseases.

Reduced BDNF levels have been reported in some psychiatric disorders and neurodegenerative diseases such as depression and Alzheimer’s disease, strongly indicating that BDNF inducers have beneficial effects in these diseases. Further, it has also been reported that higher *BDNF* expression levels in the brain are associated with slower cognitive decline^46^. Our current screening assay is useful for identifying *Bdnf* inducers from crude extracts prepared from herbal medicines and chemical compound libraries, and possibly also from natural foods. Therefore, this assay could be used to develop cognitive stabilisers in the future, which could be consumed as part of a daily diet to suppress cognitive impairment related to aging via increased *Bdnf* expression in the brain. Taken together, our screening assay could contribute to identifying candidate drugs for improving symptoms in neural diseases as well as protective agents of age-related cognitive decline, at the early phase of drug screening.

## Methods

### Reagents and libraries

A68930 hydrochloride, (R)-propylnorapomorphine hydrochloride, and cabergoline were purchased from Santa Cruz Biotechnology (Dallas, TX, USA). D-APV, FK506 monohydrate, actinomycin D, L-(−)-norepinephrine (+)-bitartrate salt monohydrate, serotonin, nicardipine, U0126, KN93, oleoyl-L-α-lysophosphatidic acid sodium salt, and Ki 16425 were purchased from Sigma-Aldrich (St. Louis, MO, USA). Neurotransmitter libraries (dopaminergic, adrenergic, serotonergic, cholinergic, histaminergic, metabotropic glutamatergic, and GABAergic) were purchased from Enzo Life Sciences, Inc. (Farmingdale, NY, USA). Ginsenoside kit (Rb1, Rb2, Rc, Rd, Re, Rf, Rg1, and Rg2), used in Fig. 5a, was purchased from Extrasynthese (Genay Cedex, France). Ginsenoside Rb1, Rc, Rd, Re, and Rg1, used in Fig. 5d, were purchased from LKT Laboratories, Inc. (St. Paul, MN, USA). Herbal extract and herbal medicine-derived compound libraries were kindly donated by the Institute of Natural Medicine, University of Toyama (Toyama, Japan). Each herbal extract used in this study was obtained using a standard method described as follows: each herbal medicine (purchased from Tochimoto Tenkaido (Osaka, Japan)) was extracted in water (10-times volume of herbal medicine) at 100 °C for 50 min, evaporated under reduced pressure, and freeze‐dried to obtain a powder extract. Each herbal extract was redissolved in water.

### Animals

All animal care and experiments were approved by the Animal Experiment Committee of the University of Toyama (Authorization No. S-2010 MED-51, A2011PHA-18, and A2014PHA-1) and Takasaki University of Health and Welfare (Authorization No. 1733 and 1809), and were performed in accordance with the Guidelines for the Care and Use of Laboratory Animals of the University of Toyama and Takasaki University of Health and Welfare. Mice were housed under standard laboratory conditions (12 h-12 h/light-dark cycle, room temperature at 22 ± 2 °C) and had free access to food and water. The generation of *Bdnf-Luc* mice has been described previously^19^. Wild-type littermates were used as control animals (Fig. 1b,c).

### Primary cultures

Primary cultures of *Bdnf-Luc* mouse cortical cells were prepared from transgenic mice at embryonic day 16.5, as described previously^19^. The cerebral cortex was isolated from each embryonic brain, and tissue lysates were briefly prepared from the remaining brain using a passive lysis buffer (Promega, Madison, WI, USA). Next, luciferase activity of each lysate was measured using a luminometer, and transgenic brains were selected on the basis of luciferase activity. Dissociated cells were seeded at 7.6 × 10^4^ cells and cultured in poly-L-lysine-coated 96-well culture plates (Greiner Bio-One, Kremsmünster, Austria) with neurobasal medium (Thermo Fisher Scientific, Waltham, MA, USA) containing B27 supplement (Thermo Fisher Scientific), 2 μg/mL gentamicin (Thermo Fisher Scientific), and 0.5 mM glutamine (Thermo Fisher Scientific). In Supplementary Figure S4a, dissociated cells were seeded at 1.8 × 10^6^ cells and cultured in poly-L-lysine-coated 6-well plates (AGC Techno Glass, Shizuoka, Japan) for RT-PCR. Half the culture medium was replaced with fresh medium every 3 days.

Primary cultures of rat cortical cells were prepared from Sprague-Dawley rats at embryonic day 17 (Japan SLC, Shizuoka, Japan), as described previously^19^. Dissociated cells were seeded at 1.8 × 10^6^ cells and cultured in poly-L-lysine-coated 6-well plates (AGC Techno Glass) for RT-PCR, or at 8 × 10^5^ cells in poly-L-lysine-coated 12-well plates (AGC Techno Glass) for a reporter assay with neurobasal medium, described above. For immunostaining, cells were seeded at 8 × 10^5^ cells and cultured on poly-L-lysine-coated coverslips of 18 mm in diameter (Matsunami Glass Ind., Ltd., Osaka, Japan) in 12-well plates (AGC Techno Glass). Half the culture medium was replaced with fresh medium every 3 days.

Primary cultures of *Bdnf-Luc* mouse cerebellar granule cells were prepared from transgenic mice at postnatal day 7, as described previously^33^. Dissociated cells were seeded at 7.6 × 10^4^ cells and cultured in a poly-L-lysine-coated 96-well culture plate with neurobasal medium, described above. At 3 and 6 DIV, half the culture medium was replaced with fresh medium.

### Measuring luciferase activity

Primary cultures of *Bdnf-Luc* mouse cortical cells at 13 DIV were treated with 25 mM KCl (for 6 h), a series of compounds (for 6 h), or herbal extracts (6, 24, or 48 h). Primary cultures of *Bdnf-Luc* mouse cerebellar granule cells at 7 DIV were treated with 25 mM KCl (for 6 h). Luciferase activity of each well was measured using the Steady-Glo Luciferase Assay System (Promega) with a Glo-Max Navigator Microplate Luminometer (Promega), according to the manufacturer’s instruction. White adhesive seal was added to the bottom of the microplate (Perkin Elmer, Waltham, MA, USA) to make it opaque before the measurement of luciferase activity.

### RT-PCR

Total RNA from cultured rat cortical cells was prepared using an ISOSPIN Cell & Tissue RNA kit (Nippongene, Tokyo, Japan). One microgram of purified total RNA was reverse-transcribed into cDNA, as described previously^19^. Real-time PCR was performed using SYBR Select Master Mix (Thermo Fisher Scientific), according to the manufacturer’s instructions. PCR thermal profiles included an initial heating at 50 °C for 2 min then at 95 °C for 2 min, followed by 45 cycles of denaturation at 95 °C for 45 s, annealing at 57 °C for 45 s, and extension at 72 °C for 1 min. Fold-change values were calculated by the ^ΔΔ^Ct method to determine relative gene expression. Primer sequences were as follows: rat *Gapdh*-forward, 5′-ATCGTGGAAGGGCTCATGAC-3′; rat *Gapdh*-reverse, 5′-TAGCCCAGGATGCCCTTTAGT-3′; rat total *Bdnf*-forward, 5′-CTGGAGAAAGTCCCGGTATCAA-3′; rat total *Bdnf*-reverse, 5′-TTATGAACCGCCAGCCAATTCTCTT-3′; rat *Bdnf* exon I-forward, 5′-CAAACAAGACACATTACCTTCCAGC-3′; rat *Bdnf* exon II-forward, 5′-AGCCAGCGGATTTGTCCGA-3′; rat *Bdnf* exon III-forward, 5′-CTCCCCGAGAGTTCCG-3′; rat *Bdnf* exon IV-forward, 5′-GGAAATATATAGTAAGAGTCTAGAACCTTGG-3′; rat *Bdnf* exon V-forward, 5′-CTCTGTGTAGTTTCATTGTGTGTTCG-3′; rat *Bdnf* exon VI-forward, 5′-GACCAGGAGCGTGACAAC-3′; rat *Bdnf* exon VII-forward, 5′-AAAGGGTCTGCGGAACTCCA-3′; rat *Bdnf* exon VIII-forward, 5′-GACTGTGCATCCCAGGAGAA-3′; rat *Bdnf* exon IXA-forward, 5′-GGTCTGAAATTACAAGCAGATGGG-3′; and rat *Bdnf* exon IX common-reverse, 5′-ACGTTTGCTTCTTTCATGGGCG-3′. To detect each exon-specific *Bdnf* transcript, a 5′ exon-specific forward primer and exon IX common reverse primer were used. In Supplementary Figure S4a, total RNA from cultured *Bdnf-Luc* cortical cells was prepared and RT-PCR was performed as described above. Expression levels of *Bdnf* and *Luciferase* mRNA were analysed by generating standard curves using cDNA dilution series. Primer sequences were as follows: mouse *Bdnf*-forward, 5′-AAGGACGCGGACTTGTACAC-3′; mouse *Bdnf*-reverse, 5′-CGCTAATACTGTCACACACGC-3′; *Luciferase*-forward, 5′-CGAGTACTTCGAGATGAGCG-3′; and *Luciferase*-reverse, 5′-CGTTGTAGATGTCGTTAGCTGG-3′.

### Microarray analysis

Microarray was performed according to previous studies^19,47^ using a GeneChip Rat Genome 230 2.0 Array and 3′ IVT Express Kit (Affymetrix, Santa Clara, CA, USA). Primary cultures of rat cortical cells at 13 DIV were treated with 500 μg/mL GIN for 3 h, and total RNA was extracted using the RNeasy Mini Kit and QIAshredder (QIAGEN, Hilden, Germany) for microarray analysis.

### Reporter assay

For reporter assays, half the culture medium was replaced with fresh medium at 3, 6, and 10 DIV, then DNA transfection was performed by the calcium/phosphate DNA co-precipitation method at 11 DIV. One hour before the addition of calcium/phosphate/DNA mixture, conditioned medium was removed and kept in a 10% CO_2_ incubator at 37 °C, and serum-free Dulbecco’s modified Eagle medium (DMEM) with high glucose (Invitrogen, Catalog No.12100046) without antibiotics that had been pre-warmed in a 10% CO_2_ incubator at 37 °C was added to the cultured cells. Calcium/phosphate/DNA precipitates were prepared by mixing 200 μL of plasmid DNA (16 μg, pGL4.12-*Bdnf-pIV*:phRL-TK(int−) = 10:1) in 250 mM CaCl_2_ solution with an equal volume of 2 × HEPES buffered saline (42 mM HEPES [pH 7.03], 274 mM NaCl, 9.5 mM KCl, 2.67 mM Na_2_HPO_4_, and 15 mM glucose) and then incubating at room-temperature for 15 min. Next, 95 μL of mixture was added to the cultured cells and incubated in a 10% CO_2_ incubator at 37 °C for 15 min. Cells were then washed twice with serum-free D-MEM with high glucose in the absence of any antibiotics that had been pre-warmed in 10% CO_2_ incubator at 37 °C. Conditioned medium was then returned to the cells. Detailed information on the plasmid DNA for measuring the activity of *Bdnf-pIV* was described previously^19^. Two days after DNA transfection, cells were treated with vehicle or GIN extract at a final concentration of 500 μg/mL for 6 h, and firefly and *Renilla* luciferase activities were measured using the Dual-Luciferase Assay System (Promega), according to the manufacturer’s instruction. Firefly luciferase activity was normalised to *Renilla* luciferase activity.

### Immunostaining

At 13 DIV, primary cultures of rat cortical cells were treated with vehicle or GIN extract at a final concentration of 500 μg/mL for 30 min. Immunostaining was then performed using an antibody against microtubule-associated protein 2 (MAP2) (Catalog No. M4403; Sigma-Aldrich) or phosphorylated CREB at serine 133rd (Catalog No. 9198; Cell Signaling Technology, Inc., Danvers, MA, USA), or antiserum against CRTC1 (kindly donated by Dr. Hiroshi Takemori, Graduate School of Engineering, Gifu University, Gifu, Japan), as described previously^19^. Nuclei were counter-stained with 300 nM 4′, 6-diamidino-2-phenylindole (Invitrogen). Confocal fluorescent images were obtained using an LSM 700 confocal microscope (Carl Zeiss, Oberkochen, Germany). The number of MAP2 and phospho-CREB positive neurons or MAP2 and nuclear localized CRTC1 (estimated as described previously^19,22^) positive neurons were counted, and the percentage of the neurons with phospho-CREB or nuclear localized CRTC1 was calculated.

### Statistics

All data are presented as mean ± the standard error of the mean (S.E.M.). Statistical analyses were performed using Prism 7 software (GraphPad, San Diego, CA, USA). Detailed information is shown in each figure legend.

## Supplementary information

Supplementary information

Supplementary Tables
