# Supplementary material for: Screening inducers of neuronal BDNF gene transcription using primary cortical cell cultures from BDNF-luciferase transgenic mice
Source: Sci Rep. 2019 Aug 14;9:11833. doi: 10.1038/s41598-019-48361-4 (PMC6694194; doi:10.1038/s41598-019-48361-4)

Supplementary Information

Screening inducers of neuronal BDNF gene transcription using primary cortical cell cultures from BDNF-luciferase transgenic mice

Mamoru Fukuchi[1,2,*], Yui Okuno[2], Hironori Nakayama[2], Aoi Nakano[2], Hisashi Mori[3], Satoru Mitazaki[1,4], Yuka Nakano[1], Kazufumi Toume[5], Michiko Jo[6], Ichiro Takasaki[7], Kazuki Watanabe[8], Naotoshi Shibahara[6], Katsuko Komatsu[5], Akiko Tabuchi[2], and Masaaki Tsuda[2]

[1]Laboratory of Molecular Neuroscience, Faculty of Pharmacy, Takasaki University of Health and Welfare, 60 Nakaorui-machi, Takasaki-shi, Gunma 370-0033, Japan

[2]Department of Biological Chemistry, Graduate School of Medicine and Pharmaceutical Sciences, University of Toyama, 2630 Sugitani, Toyama-shi, Toyama 930-0194, Japan

[3]Department of Molecular Neuroscience, Graduate School of Medicine and Pharmaceutical Sciences, University of Toyama, 2630 Sugitani, Toyama-shi, Toyama 930-0194, Japan

[4]Laboratory of Forensic Toxicology, Faculty of Pharmacy, Takasaki University of Health and Welfare, 60 Nakaorui-machi, Takasaki-shi, Gunma 370-0033, Japan

[5]Division of Pharmacognosy, Institute of Natural Medicine, University of Toyama, 2630 Sugitani, Toyama-shi, Toyama 930-0194, Japan

[6]Division of Kampo Diagnostics, Institute of Natural Medicine, University of Toyama, 2630 Sugitani, Toyama-shi, Toyama 930-0194, Japan

[7]Department of Pharmacology, Graduate School of Science and Engineering, University

of Toyama, 3190 Gofuku, Toyama-shi, Toyama 930-8555, Japan

[8]Laboratory of Natural Medicines, Faculty of Pharmacy, Takasaki University of Health and Welfare, 60 Nakaorui-machi, Takasaki-shi, Gunma 370-0033, Japan

[*]Corresponding author: Mamoru Fukuchi

Laboratory of Molecular Neuroscience, Faculty of Pharmacy, Takasaki University of Health and Welfare, 60 Nakaorui-machi, Takasaki-shi, Gunma 370-0033, Japan

Tel.: +81-27-352-1180 (ext. 8317); Fax: +81-27-352-1118; E-mail: fukuchi@takasaki-u.ac.jp

Supplementary Tables

Table S1. List of compounds in neurotransmitter libraries.

Table S2. Library of herbal medicine extracts.

Table S3. Library of herbal medicine-derived compounds.

Table S4. Up-regulated (a) and down-regulated (b) transcripts after GIN treatment.

Supplementary Figure Legends

Figure S1. Screening activators of *Bdnf* transcription from neurotransmitter libraries.

Representative results obtained using commercially available adrenergic (a), serotonergic (b), cholinergic (c), histaminergic (d), metabotropic glutamatergic (e), and GABAergic (f) libraries. Each compound was added into *Bdnf-Luc* cortical cells at 13 DIV at a final concentration of 10, 100, or 1000 nM, and luciferase activity was measured in each well

6 h after addition. Arrowheads show active compounds (that increased luciferase activity by more than 2-fold). For compound names, see Supplementary Table S1.

Figure S2. Effect of active compounds identified from neurotransmitter libraries on expression of endogenous *Bdnf* mRNA in primary cultures of rat cortical cells.

(a-d) At 13 DIV, cultured rat cortical cells were treated with propylnorapomorphine (a), cabergoline (b), norepinephreine (c), and serotonin (d) at the indicated concentrations for 1 h, then total RNA was prepared for RT-PCR analysis. Means ± SEM (n = 3), $p < 0.05$, ** $p < 0.01$, and *** $p < 0.001$ vs. 0 nM (one-way ANOVA with Dunnett's multiple comparisons test). (e) Relationship between changes in endogenous *Bdnf* mRNA expression levels (Fig. 2b and Supplementary Fig. S2a-d) and those in luciferase activity (Fig. 2a and Supplementary Fig. S1a-b) was analyzed using Pearson's correlation coefficient test.

Figure S3. Effect of active herbal extracts on expression of endogenous *Bdnf* mRNA in primary cultures of rat cortical cells.

At 13 DIV, cultured rat cortical cells were treated with extracts prepared from Zanthoxyli Piperiti Pericarpium (a; No. 76), Ginseng Radix (b; No. 9), Polygoni Multiflori Radix (c; No. 50), Panacis Notoginseng Radix (d; No. 54), Rhei Rhizoma (e; No. 79), Ephedrae Herba (f; No. 110), Alpiniae Officinari Rhizoma (g; No. 114), Sinomeni Caulis et Rhizoma (h; No. 112), and Uncariae Uncis Cum Ramulus (i; No. 80)) for 3 h, 24 h, or 48 h. Total RNA was prepared at the indicated times. Means ± SEM (n = 3), ** $p < 0.01$, ***

$p < 0.001$, and **** $p < 0.0001$ vs. water at the same time point (two-way ANOVA with Tukey's multiple comparisons test).

Figure S4. Effect of the transcription inhibitor actinomycin D on *Bdnf* and *Luciferase* mRNA expression and changes in luciferase activity in cultured *Bdnf-Luc* cortical cells. (a) *Bdnf-Luc* cortical cells were seeded into a 6-well culture plate and cultured for 13 days. Cells were then treated with 10 μg/mL actinomycin D (ActD), and total RNA was prepared at the indicated time. Changes in *Bdnf* (left) and *Luciferase* (right) mRNA expression were examined by RT-PCR. Means ± SEM (n = 3), ** $p < 0.01$, *** $p < 0.001$, **** $p < 0.0001$ vs. 0 h (one-way ANOVA with Dunnett's multiple comparisons test). (b) *Bdnf-Luc* cortical cells were seeded into a 96-well culture plate and cultured for 13 days. Then, cells were treated with 10 μg/mL ActD, and luciferase activity in each well measured at the indicated time. Means ± SEM (n = 3-6). (c) Changes in luciferase activity in the absence or presence of ActD. At 13 DIV, *Bdnf-Luc* cortical cells seeded into 96-well culture plates were treated with DMSO (vehicle) or ActD. Ten minutes after the addition of DMSO or ActD, cells were treated with 25 mM KCl, 1 μM norepinephrine, or 500 μg/mL GIN, and luciferase activity was measured 6 h after treatment. Means ± SEM (n = 6), ** $p < 0.01$, *** $p < 0.001$, **** $p < 0.0001$ vs. vehicle/water, †† $p < 0.01$, †††† $p < 0.0001$ vs 25 mM KCl, 1 μg/mL norepinephrine, or 500 μg/mL GIN without ActD (two-way ANOVA with Tukey's multiple comparisons test).

Figure S5. Effect of eluate fractions prepared from water extract of Ginseng Radix on

luciferase activity in primary cultures of *Bdnf-Luc* cortical cells.

(a) Ginseng Radix was extracted twice with hot water, and passed through a Diaion HP-20 (Mitsubishi-Chemical, Tokyo, Japan) column chromatography to obtain eluate fractions. Here, we used a MeOH eluate fraction with enriched ginsenosides. (b) Thin-layer chromatography (TLC) of the MeOH eluate fraction. TLC was carried out on precoated Kieselgel 60 F254 (0.25 mm thick, Merck, Darmstadt, Germany) plate, and spots were visualized by spraying the plates with 10% H_2SO_4 (aq) followed by heating. Solvent: $CHCl_3$-MeOH-H_2O (20:10:1), MeOH: MeOH eluate fraction, Rb1: ginsenoside Rb1, Re: ginsenoside Re, Rg1: ginsenoside Rg1. (c) At 13 DIV, cultured *Bdnf-Luc* cortical cells were treated with different concentrations of water extract (left) or MeOH eluate fraction, and luciferase activity was measured in each well 6 h after addition. Means ± SEM (n = 4-6), **** $p < 0.0001$ vs. 0 μg/mL (one-way ANOVA with Dunnett's multiple comparisons test).

Figure S6. Screening activators of *Bdnf* transcription from a herbal medicine-derived compound library.

Representative result obtained using a herbal medicine-derived compound library. Each compound was added into *Bdnf-Luc* cortical cells at 13 DIV at a final concentration of 10, 50, or 100 μM, and luciferase activity was measured in each well at 6 h after addition.

Figure S7. Measuring luciferase activity of cultured cerebellar granule cells prepared from *Bdnf-Luc* mice using 96-well culture plates.

Primary cultures of cerebellar granule cells were prepared from *Bdnf-Luc* mice at postnatal day 7. Cells were cultured in a 96-well culture plate, and half the culture medium was replaced with fresh medium at 3 and 6 DIV. At 7 DIV, DMSO (vehicle) or nicardipine (Nica, final concentration; 5 μM) was added to cells 10 min before the addition of KCl. Luciferase activity was measured in each well at 6 h after the addition of KCl. Means ± SEM (n = 16), **** $p < 0.0001$ vs. 5 mM KCl, †††† $p < 0.0001$ vs 25 mM KCl in the absence of nicardipine (one-way ANOVA with Dunnett's multiple comparisons test).

<u>Figure S8. Full image of TLC.</u>

A full image corresponding the cropped TLC image shown in Supplementary Figure S5 (b). This picture has not been processed at all.

Supplementary Figure S1

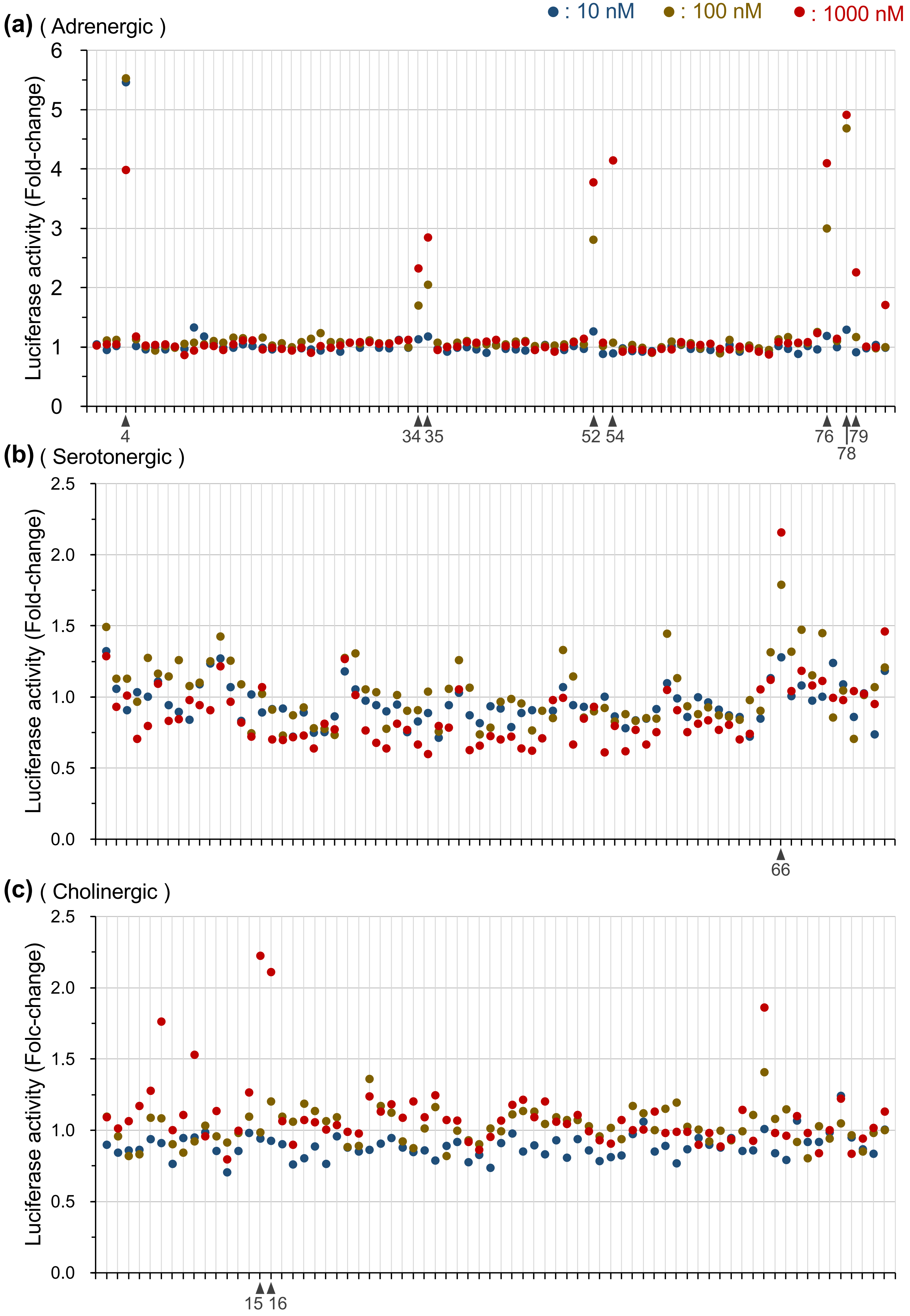

Supplementary Figure S1

(d) (Histaminergic)

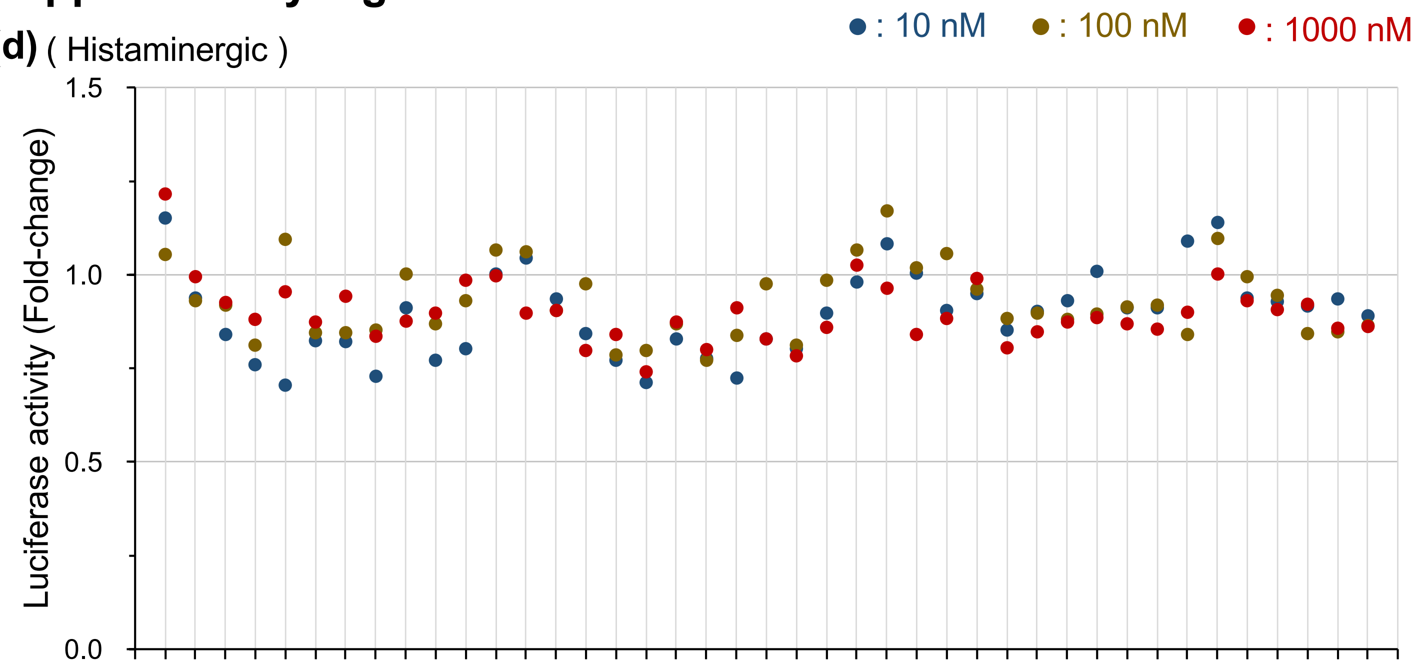

(e) (Metabotropic glutamatergic)

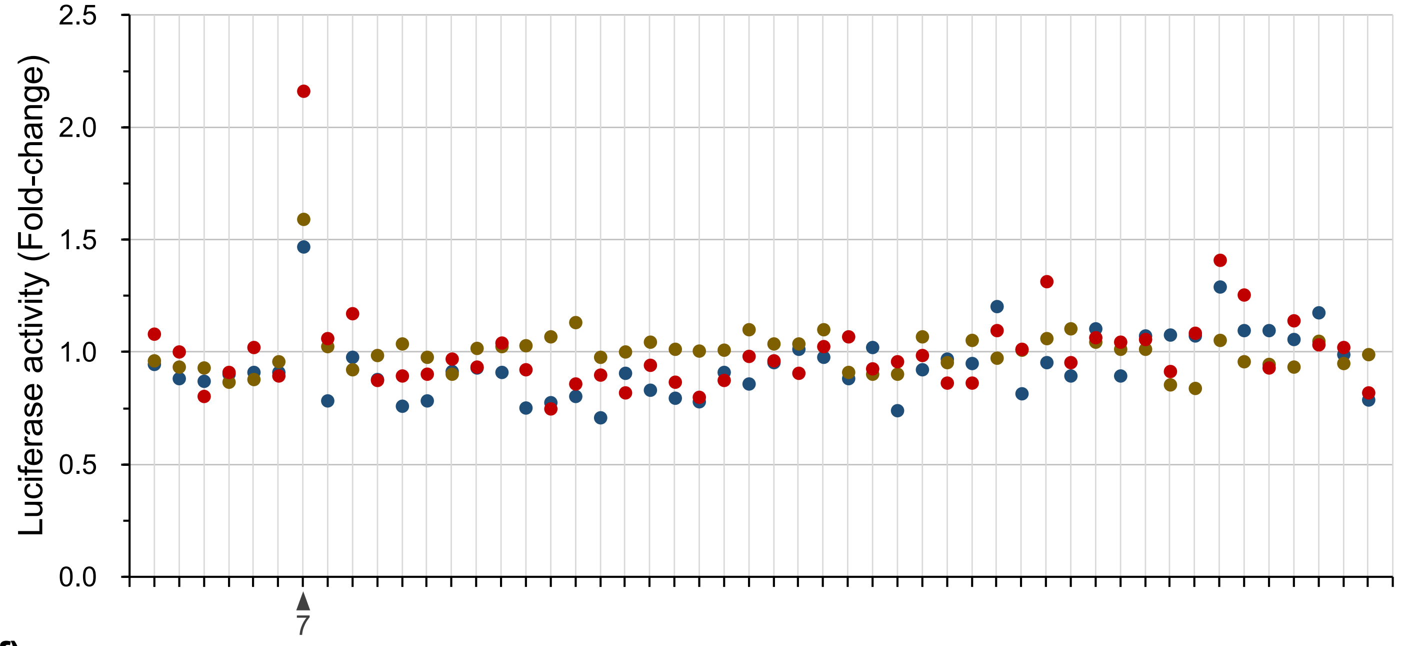

(f) (GABAergic)

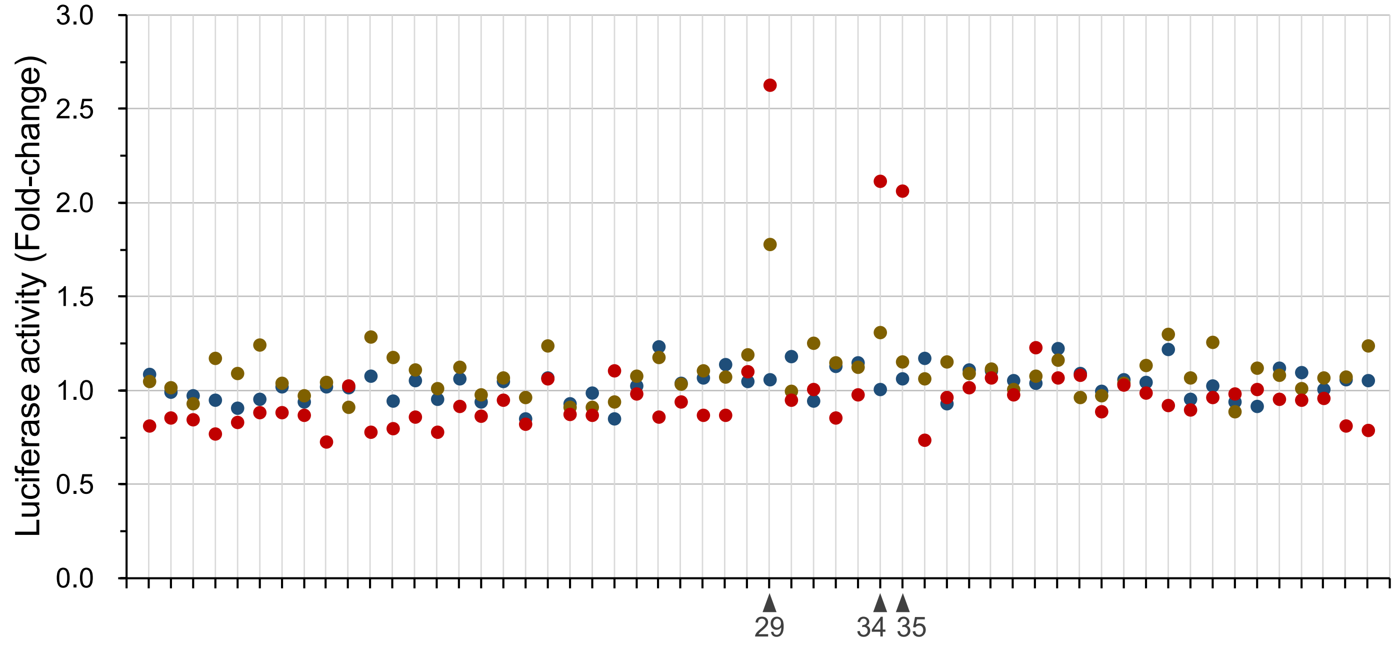

Supplementary Figure S2

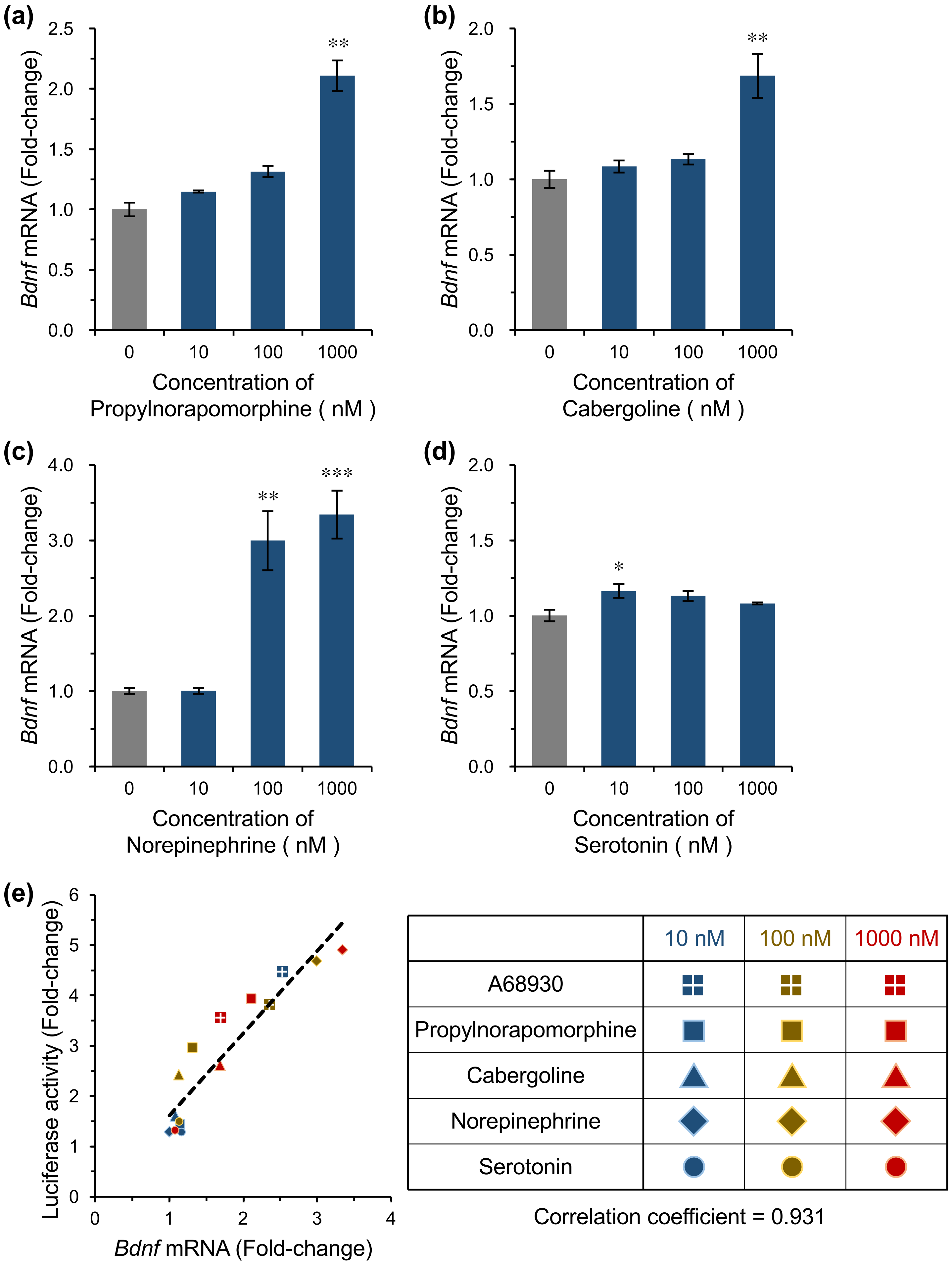

Supplementary Figure S3

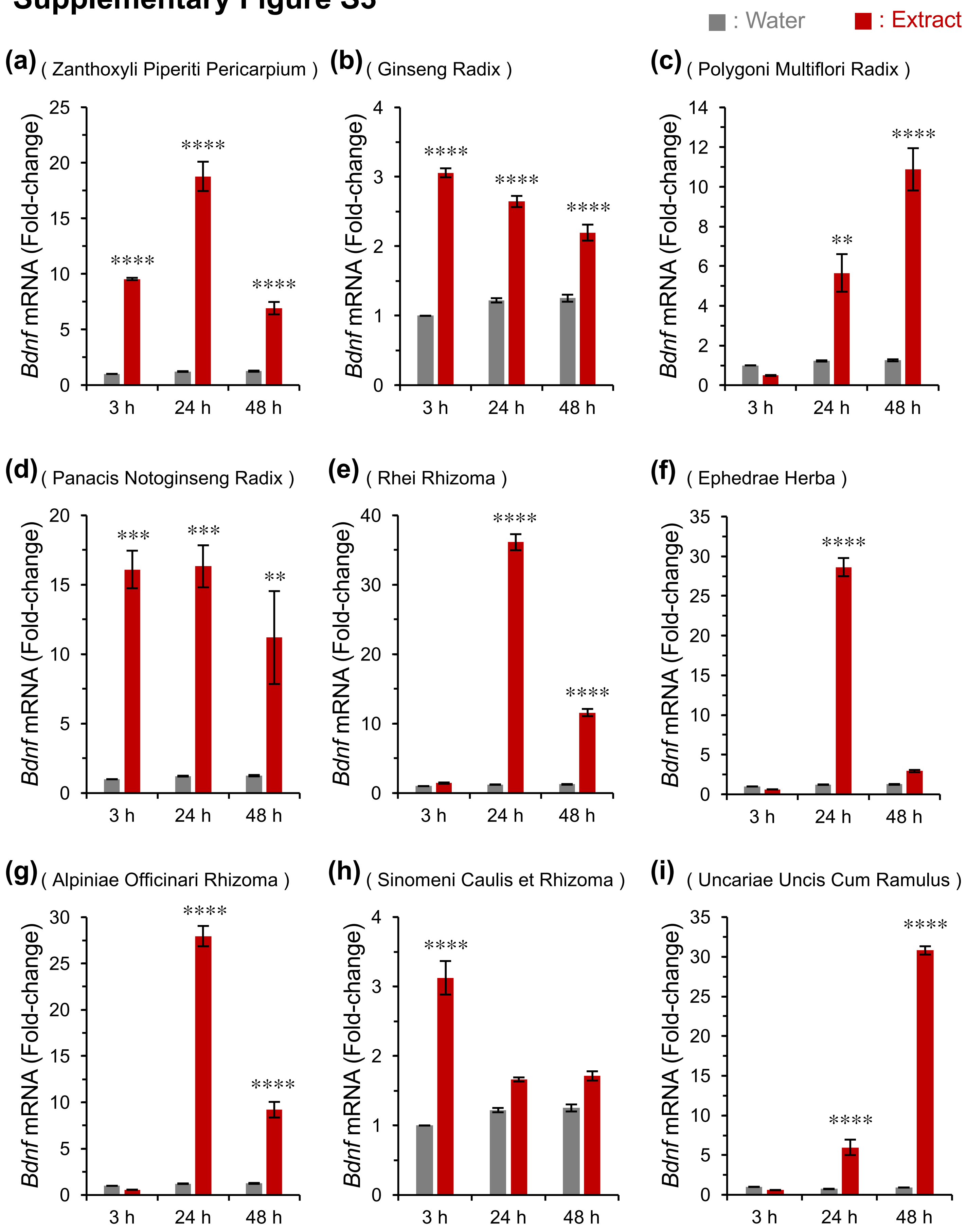

Supplementary Figure S4

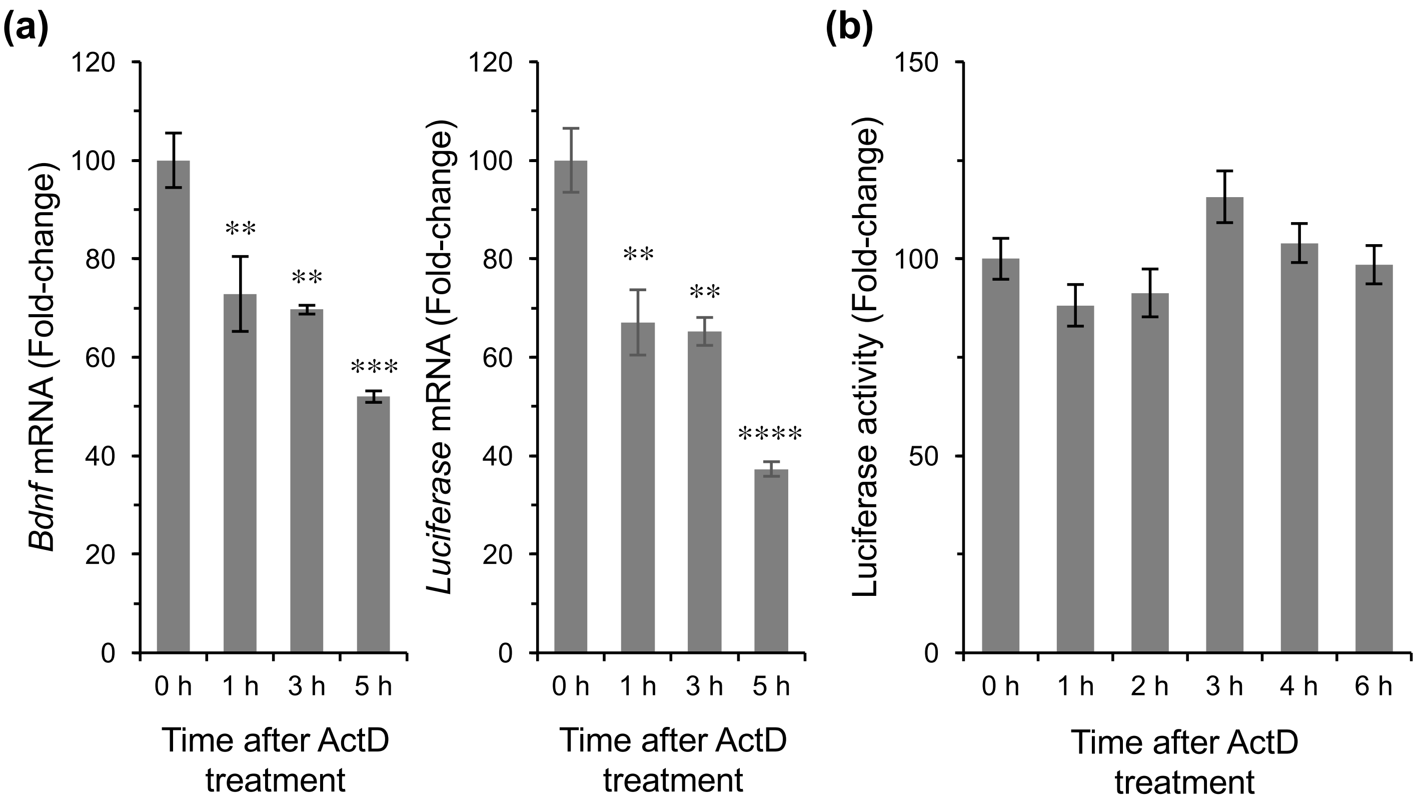

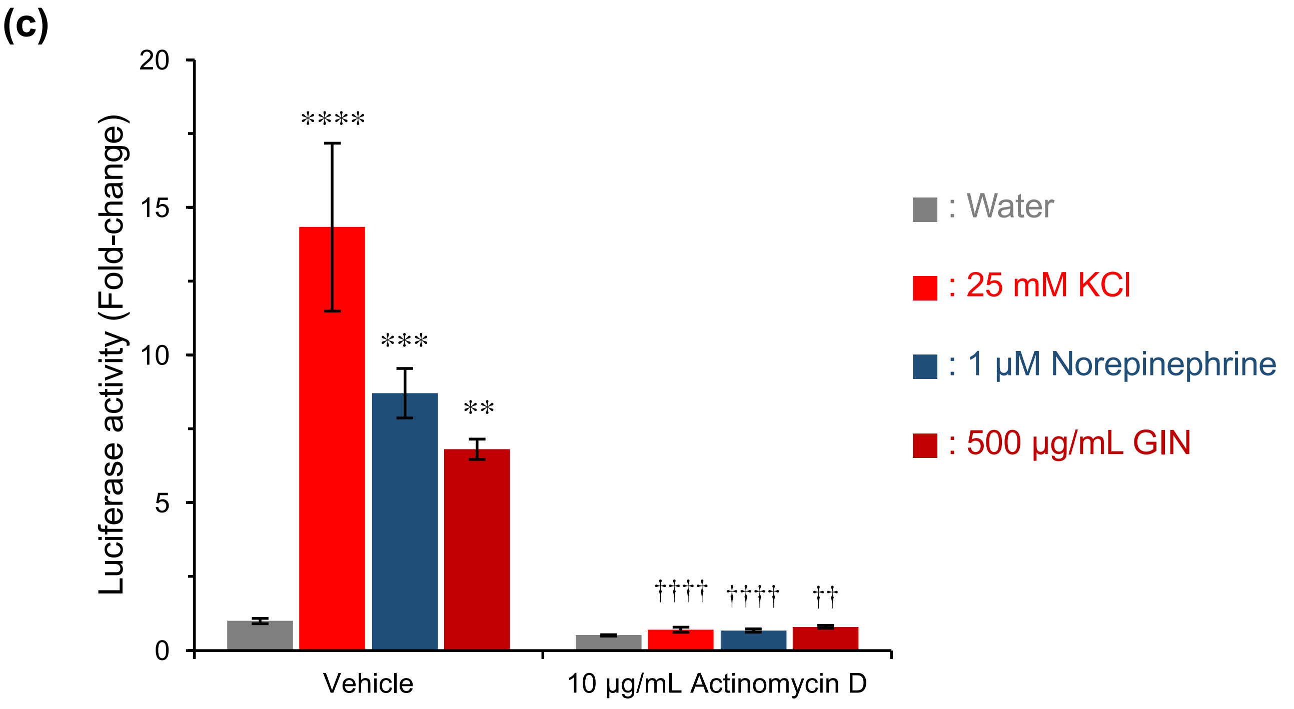

Supplementary Figure S5

(a)

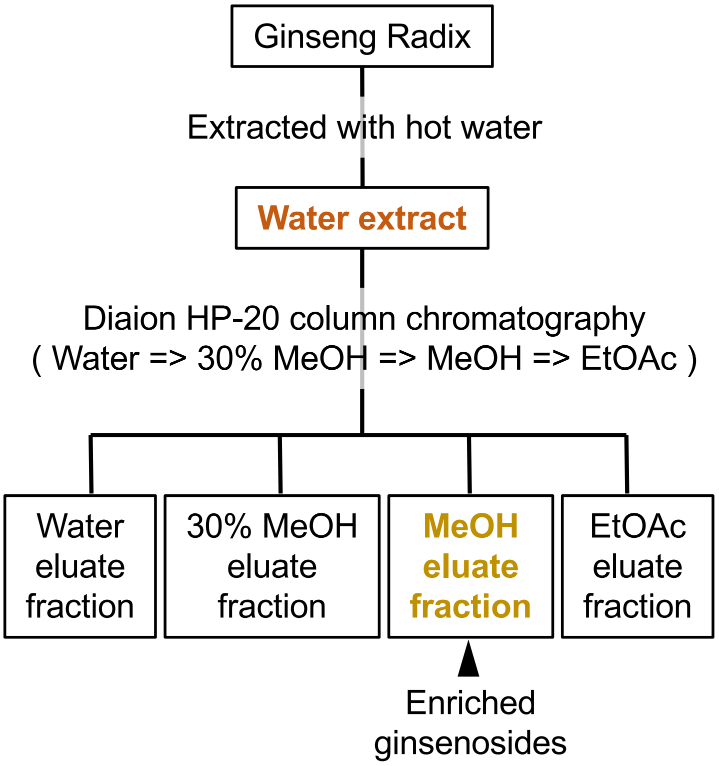

(b)

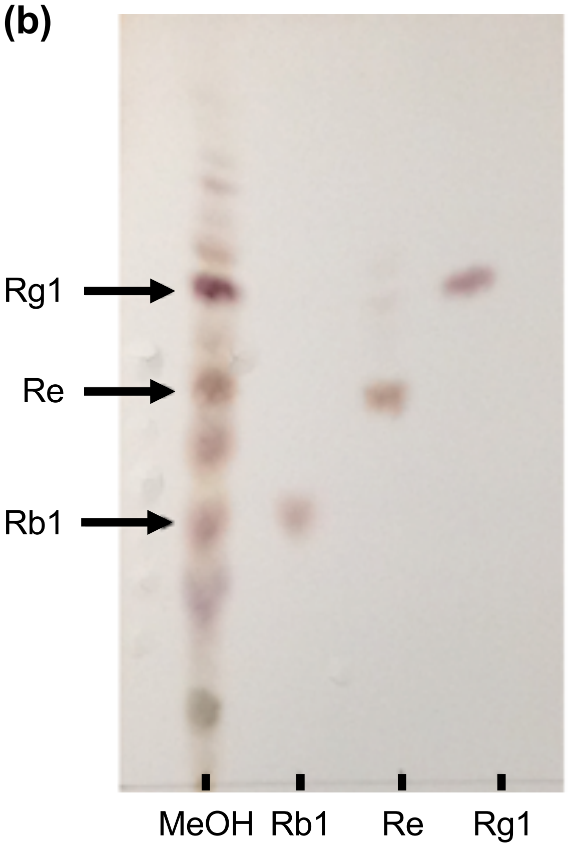

(c)

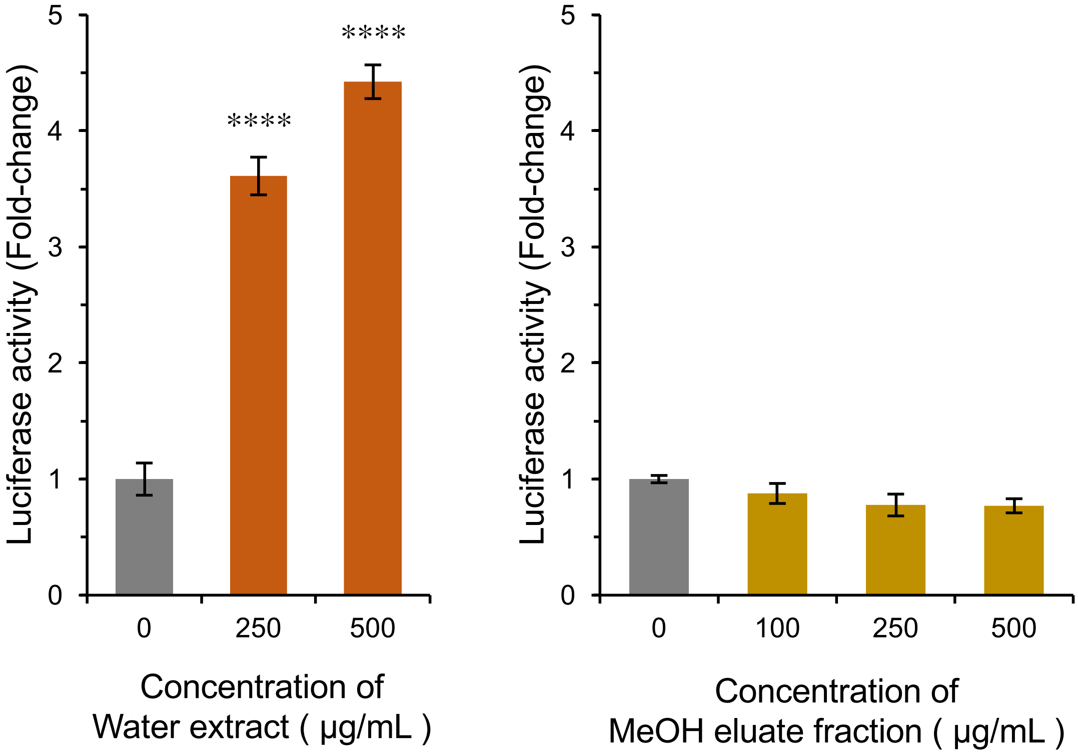

Supplementary Figure S6

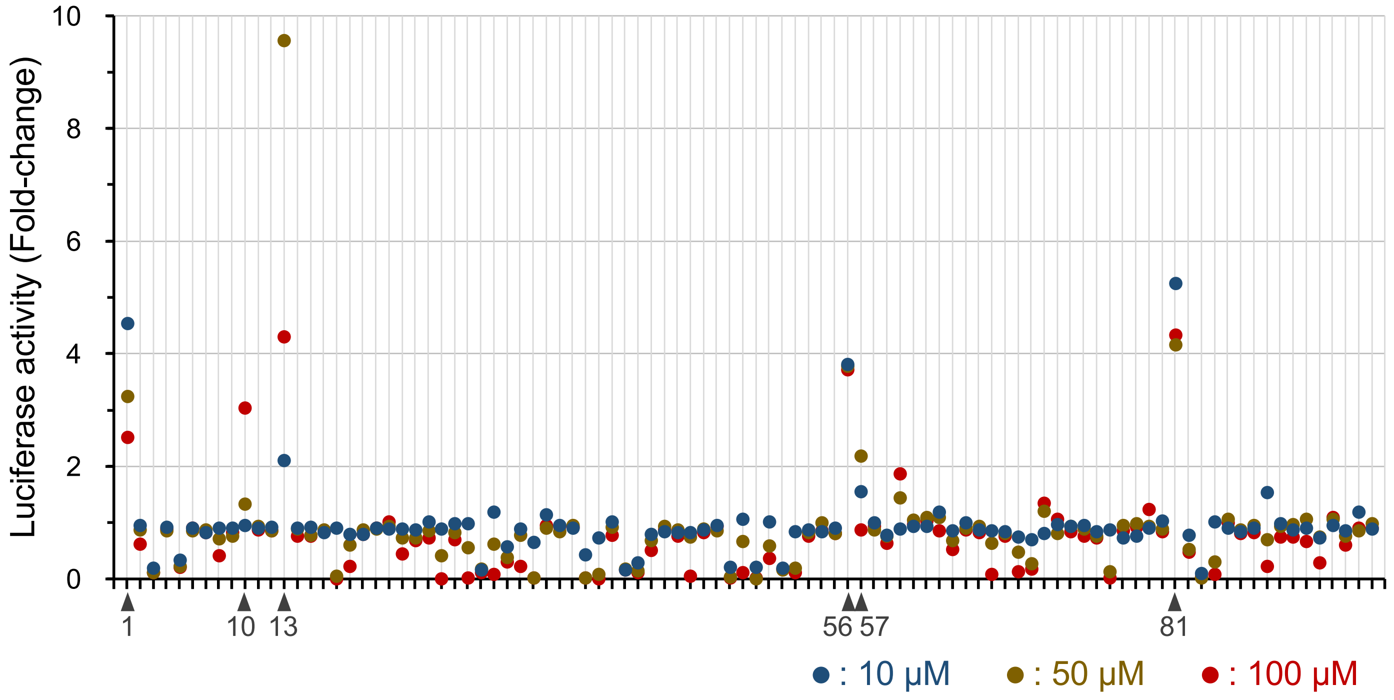

Supplementary Figure S7

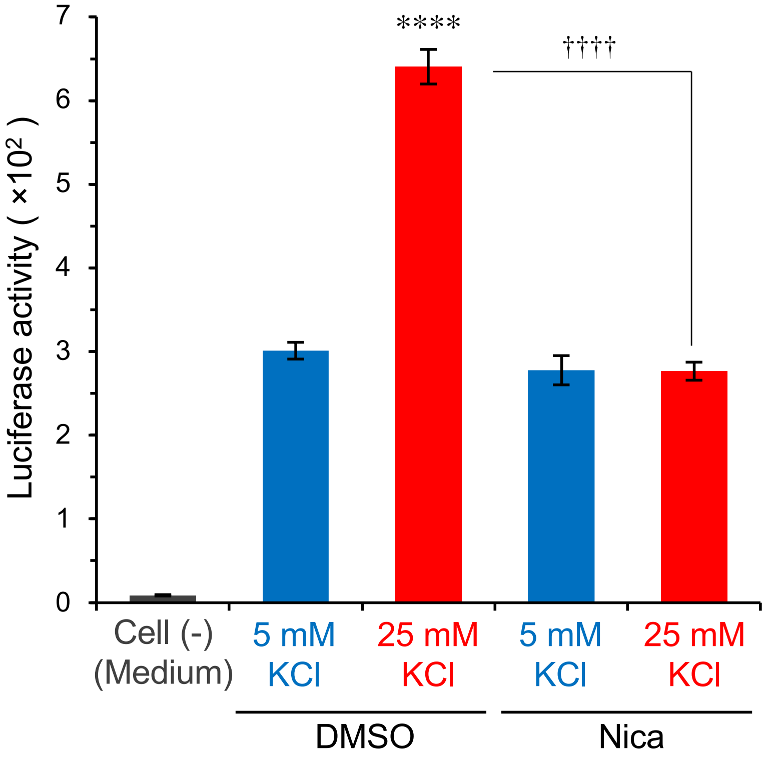

Supplementary Figure S8

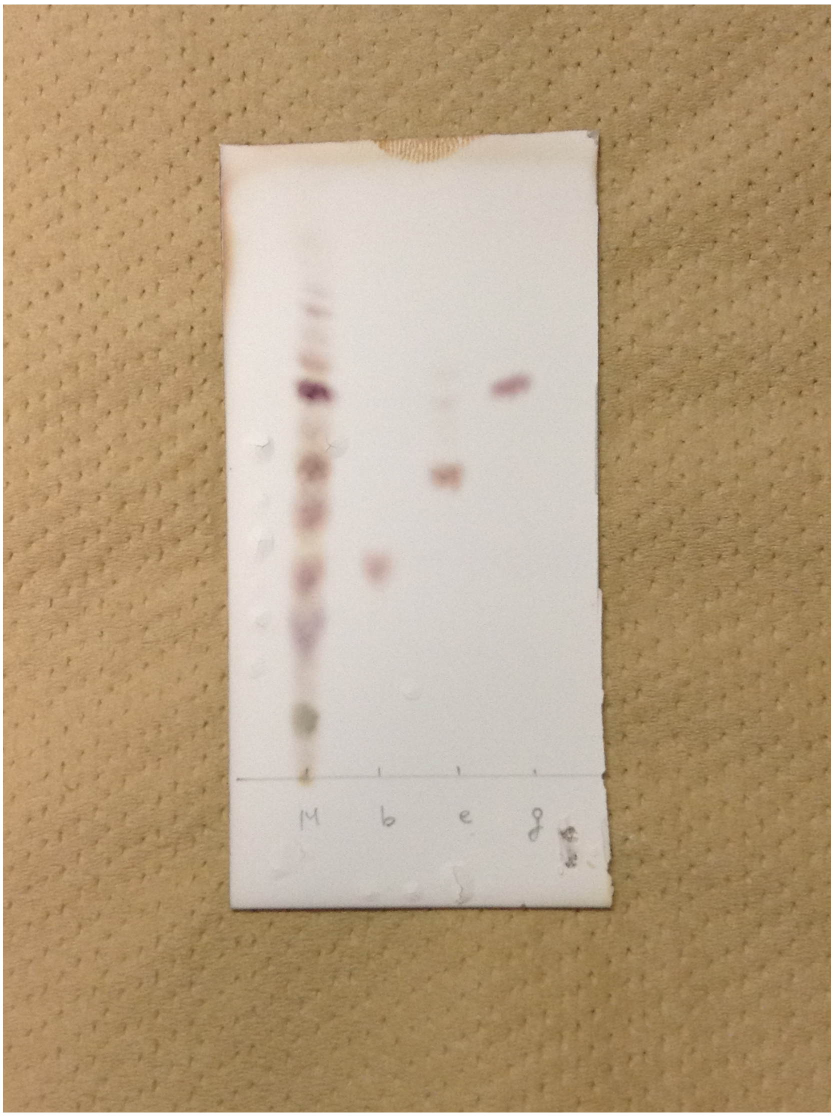

Supplement: Supplementary file 1 — Supplementary information [file 41598_2019_48361_MOESM1_ESM.pdf]
